# The impact of COVID-19 on populations living at high altitude: Role of hypoxia-inducible factors (HIFs) signaling pathway in SARS-CoV-2 infection and replication

**DOI:** 10.3389/fphys.2022.960308

**Published:** 2022-08-25

**Authors:** Christian Albert Devaux, Didier Raoult

**Affiliations:** ^1^ Aix-Marseille University, IRD, APHM, MEPHI, Marseille, France; ^2^ IHU-Méditerranée Infection, Marseille, France; ^3^ Centre National de la Recherche Scientifique, Marseille, France

**Keywords:** COVID-19, hypoxia, hypoxia-inducible factors, Nrf2, TRPA1, curcumin

## Abstract

Cases of coronavirus disease 2019 (COVID-19) have been reported worldwide. However, one epidemiological report has claimed a lower incidence of the disease in people living at high altitude (>2,500 m), proposing the hypothesis that adaptation to hypoxia may prove to be advantageous with respect to SARS-CoV-2 infection. This publication was initially greeted with skepticism, because social, genetic, or environmental parametric variables could underlie a difference in susceptibility to the virus for people living in chronic hypobaric hypoxia atmospheres. Moreover, in some patients positive for SARS-CoV-2, early post-infection ‘happy hypoxia” requires immediate ventilation, since it is associated with poor clinical outcome. If, however, we accept to consider the hypothesis according to which the adaptation to hypoxia may prove to be advantageous with respect to SARS-CoV-2 infection, identification of the molecular rational behind it is needed. Among several possibilities, HIF-1 regulation appears to be a molecular hub from which different signaling pathways linking hypoxia and COVID-19 are controlled. Interestingly, HIF-1α was reported to inhibit the infection of lung cells by SARS-CoV-2 by reducing ACE2 viral receptor expression. Moreover, an association of the rs11549465 variant of HIF-1α with COVID-19 susceptibility was recently discovered. Here, we review the evidence for a link between HIF-1α, ACE2 and AT1R expression, and the incidence/severity of COVID-19. We highlight the central role played by the HIF-1α signaling pathway in the pathophysiology of COVID-19.

## Introduction

An outbreak of severe acute respiratory syndrome was observed in Wuhan (China) in December 2019. This new disease was characterized by acute respiratory distress (ARDS), cytokine storm, and thrombotic events leading to multiple organ dysfunction syndrome (MODS) and a risk of death. One of the biggest and most life-threatening mysteries of COVID-19 is how SARS-CoV-2 cause “happy hypoxia” (Couzin-Frankel, 2020). The “happy hypoxia” or “silent hypoxia” is a clinical phase during which the arterial oxygen saturation of hemoglobin (SaO_2)_ of patients decreases (likely due to clots in small blood vessels) while patients, due to the lack of dyspnoea, describe themselves as comfortable (patients do not notice any vital signs of collapsed alveoli and air sacs) at the risk of seeing their state of health deteriorate rapidly if gone undetected for too long. Abnormally extremely low oxygen levels in the body can irreparably damage vital organs in absence of immediate ventilation (Couzin-Frankel, 2020; Brouqui et al., 2021). It has been suggested that “happy hypoxia” is caused by a combination of biological mechanisms taking place in the lungs of COVID-19 patients including microvascular thrombosis, pulmonary embolism, ventilation-perfusion mismatching in the non-injured lung, capillary flow redistribution, air sacs collapse (atelectasis), flow through intrapulmonary arteriovenous anastomoses, and normal perfusion of the relatively small fraction of injured lung (Herman et al., 2020; Nitsure et al., 2020; Rahman et al., 2021). The prevalence of “happy hypoxia” in COVID-19 patients ranges from 20% to 40% (Fuglebjerg et al., 2020; Tobin et al., 2020).

Within 28 months this infectious disease (later named COVID-19) was responsible for more than 6.25 million deaths among 518 million infected people (https://coronavirus.jhu.edu/map.html), and COVID-19 did not spare those living at high altitudes (>2,500 m) (Arias-Reyes et al., 2020; Huamanı´ et al., 2020; Xi et al., 2020; Zeng et al., 2020). However, one epidemiological report by Arias-Reyes and others, claimed a lower COVID-19 incidence in populations living at high-altitude (Arias-Reyes et al., 2020). The authors hypothesised the occurrence of a metabolic switch in hypoxic environments which could be advantageous against SARS-CoV-2 infection and/or disease severity. Most indigenous high-altitude populations live on the Andean altiplano in South America (Peru and Bolivia), the Tibetan plateaux in Asia and the Ethiopian Highlands in Africa (Julian and Moore, 2019). Although exposure to chronic hypoxia and adaptation to hypoxia is a major environmental factor that characterizes these populations, other variables (including social variables such as population density; genetic variables such as ACE2 or TMPRSS2 polymorphism; and, environmental variables such as UV radiation and humidity) could underlie a difference in susceptibility to the virus (Pun et al., 2020). Interestingly, these populations have overcome the low ambient oxygen tension via different modes of adaptation as illustrated by the fact that Andean highlanders’ haemoglobin concentration and SaO_2_ are strikingly higher than those in Tibetans (Moore et al., 2002; Beall, 2003). The apparent benefit of having been chronically exposed to hypoxia prior to SARS-CoV-2/COVID-19 could appear to be counter-intuitive with respect to the post-infection “happy hypoxia”, a pathophysiological phenomenon in which hypoxia instead increases the patient’s risk of developing severe forms of COVID-19. Furthermore, it has been reported that SARS-CoV-2 ORF3a-induced mitochondrial damage leads to the expression of hypoxia-inducible genes which aggravates viral infection and inflammatory responses (Tian et al., 2021). In fact, it is not hypoxia *per se* that could be beneficial, but the molecular mechanisms of adaptation to hypoxia transmitted from generation to generation in populations living at high altitude. There are a few arguments in favor of a beneficial role of adaptation to hypoxia in SARS-CoV-2 infection which could support the Arias-Reyes hypothesis (Arias-Reyes et al., 2020): 1) hypoxic activation of the hypoxia-inducible factor (HIF)-1α was reported to inhibit epithelial cells attaching to SARS-CoV-2 by modulating the expression of ACE2 and heparan sulfate (Prieto-Fernandez et al., 2021; Wing et al., 2021) and, 2) an association of rs11549465 (C1772T) variant of HIF-1α with COVID-19 susceptibility was described (Das et al., 2021). All this deserves to be analyzed in greater depth both by reductionist and holistic approaches.

## Hypoxia and cases of coronavirus disease 2019

The ability to maintain O_2_ homeostasis is essential for human survival. Barometric pressure is inversely proportional with altitude. The partial O_2_ (PaO_2_) pressure in the arterial blood is determined by alveolar ventilation and the alveolar-arterial O_2_ gradient. At 4,000-m altitude, every breath of air contains only 60% of the O_2_ molecules in the same breath at sea level with 90 mmHg O_2_ inhaled rather than 150 mmHg (20% O_2_) (Beall, 2007). Adaptation to hypoxia occurs through ventilation which controls the volume of air and O_2_ delivered into the lung alveoli and leads to a higher concentration of haemoglobin of erythrocytes in the bloodstream which captures O_2_ exchanged through the alveolar-capillary system (Wu et al., 2005). It was observed that hypoxia decreases pulmonary nitric oxide gas (NO) and causes vasoconstriction (Gustafsson et al., 1991). The inhalation of NO improved SaO_2_ in patients with hypoxic pulmonary vasoconstriction (Scherrer et al., 1996). Levels of NO in the lungs and plasma of Tibetans are twice those observed in other populations (Beall et al., 2012). Healthy men who climb to 3,500-m altitude experience a decrease in aldosterone resulting from a mechanism by which the potassium balance could be maintained by hyperventilation (Raf, 1991). Mountaineers who visit high altitude while normally living near sea level experience acute physiological adjustments that ensure life-sustaining oxygen delivery to the tissues despite a reduction in the partial pressure of oxygen (Bärtsch and Gibbs, 2007). Adaptation to high altitude or chronic obstructive pulmonary disease are associated with pathophysiological mechanisms similar to those encountered in sustained hypoxia (Forth and Montgomery, 2003; Prabhakar and Peng, 2004). Several genes have been considered as candidates for adaptation to hypoxia including ACE1, myoglobin, endothelial NO synthase, heme oxygenase 2, prolyl hydroxylase 2, FAM213A, lung surfactant protein D, and HIF genes (Gesang et al., 2002; Moore et al., 2004; Bigham et al., 2008; Drozdovska et al., 2009; Beall et al., 2010; Bigham et al., 2010; Simonson et al., 2010; Valverde et al., 2015; Bigham, 2016). Pregnant women living at high altitudes are at increased risk of intrauterine growth restriction and pre-clampsia (Moore et al., 2004). Moreover, Tibetan women who were homozygous or heterozygous for high SaO_2_ autosomal dominant allele had twice as many surviving children as women who were homozygous for the low SaO_2_ allele (Beall, 2007). Like humans, the animals that have lived on the Tibetan Plateau for thousands of years have had to adapt to this extreme environment. Tibetan sheep living on both highland or lowland represent ideal organisms to investigate adaptation to high altitude. A genome-wide analysis carried out on the two groups of sheep revealed selection events spanning genes involved in angiogenesis, energy production and erythropoiesis (Wei et al., 2016).

Thus, pre-existing adaption to hypoxia could be beneficial in providing better resistance to lower oxygenation found during COVID-19.

## Hypoxia-inducible factor and genes expression

**FIGURE 1:**
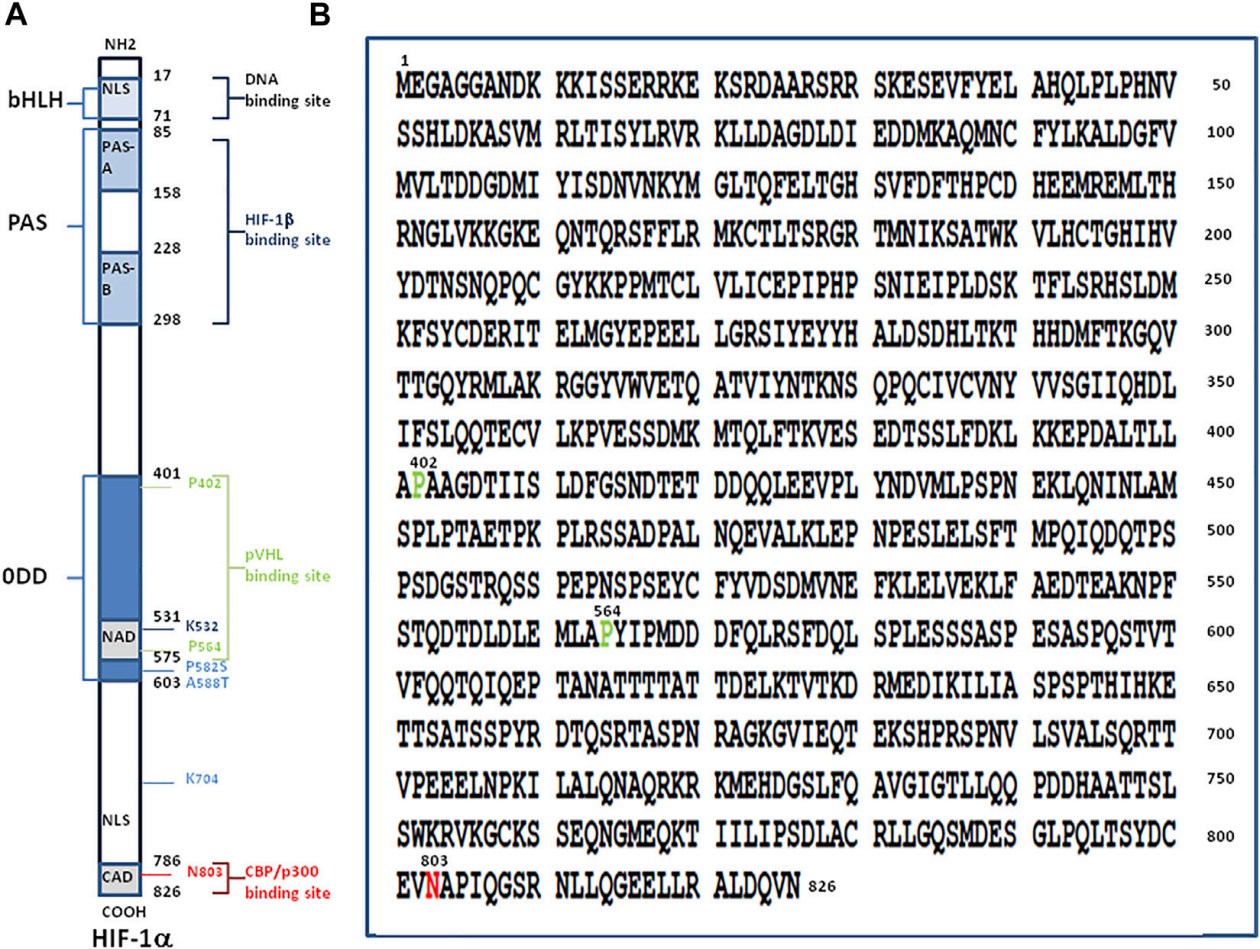
Structure of hypoxia-inducible factor, HIF-1α. **(A)** Schematic representation of the HIF-1α protein (left panel). The numbers in black indicate the first and last amino acid residues of each domain of the molecule. From the NH2-terminal to the COOH-terminal region of the protein, the main domains are: i) a N-terminal region with the basic domain (aa 17-30), a basic helix-loop-helix domain (bHLH; aa 31-71) composed of two amphipatic α-helices connected by a loop, and a nuclear localisation signal (NLS; aa 17-74); ii) a Per-AHR-ARNT-Sim homology domains (PAS including the PAS-A aa 85-158, and PAS-B aa 228-298) involved in heterodimerisation with HIF-1β; iii) an oxygen-dependent degradation domain (ODD; aa 401-603) which contains two PEST-like motifs (aa 499-518 and 581-600) rich in proline (P), glutamic acid **(E)**, serine (S) and threonine (T) (not shown); iv) a transactivation domains (TADs also termed NAD aa 531-575 and CAD aa 786-826) with the conserved residues 792-796 making contacts with the surface zinc-binding TAZ1 domain of CBP; and v) a C-terminal NLS (aa 718-721). Prolines P-402 and P-564 within the ODD and asparagine N-803 within CAD are requires for the full activation of HIF-1α. Under normoxic conditions the N-803 is hydoxylated by the FIH-1 hydroxylase and P-402 and P-564 within the ODD are hydroxylated by the prolyl hydroxylase PHD and the hydroxyproline residues become buried within the hydrophobic core of pVHL leading to ubiquitinylation of HIF-1α. The HIF-1α protein stability is increased by acetylation on lysine K-709 as well as by proline P-582 substitution (P582S) and alanine A-588 substitution (A588T). Acetylation of lysine K-532 promotes interaction with and ubiquitination by pVHL. **(B)** Amino acid sequence of HIF-1α from *Homo sapiens* (NCBI reference sequence NP8001521.1). The amino acids P-402, P-564 and N-803 are highlighted.

In the presence of an adequate supply of oxygen, the newly translated HIF-1α (a protein made up of 826 amino acids) (Figure 1), is hydroxylated at its oxygen-dependent degradation domain (ODD) and degraded by the ubiquitination proteasome system (UPS) while under hypoxic conditions HIF-1α evades the UPS and translocates into the cell nucleus where it acts as a master transcriptional regulator. The transcription factor HIF plays a major role in the O_2_ sensing system and regulates numerous downstream genes including those involved in hypoxia regulation such as erythropoietin which stimulates erythrocyte proliferation. Varying levels of the HIF gene and protein expression have been reported in people with chronic mountain sickness (Beall, 2003). HIF binds to hypoxia regulated elements (HREs) in the promoter region of hypoxia-inducible genes as an α/β heterodimer forming a complex with the histone acetyltransferases CBP/p300 (Wang et al., 1995; Kallio et al., 1998; Dames et al., 2002; Ratcliffe, 2013). The cysteine/histidine-rich 1 (CH1) domain of p300 binds to the C-terminal transactivation domain of HIF-1α and acts as a scaffold for specific folding of HIF-1α The p300 can also increase HIF-1α protein stability via Lys-709 acetylation. HIF, via a specific HRE, activates the transient receptor potential channel (TRP) ankyrin repeat (TRPA1) which leads to intracellular calcium increase, modulation of cytokine release, and cell injury (Seta et al., 2004; Hatano et al., 2012; Mori et al., 2017; Danta, 2021).

**FIGURE 2:**
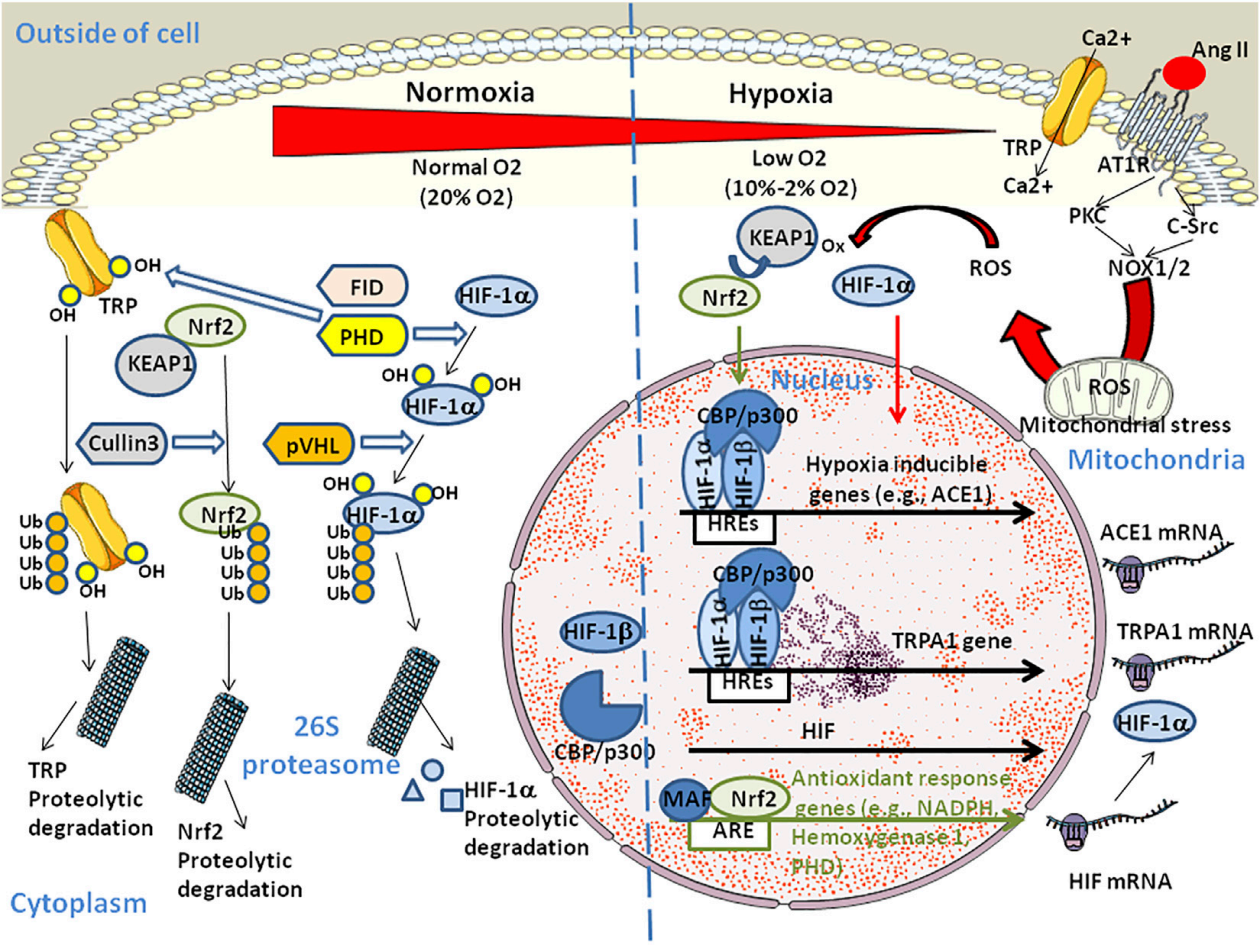
Schematic diagram illustrating the dual regulation of HIF. At high O2 concentration, HIF-1α is hydroxylated on proline by the prolyl hydroxylase PHD. The ubiquitin ligase VHL targets HIV-1α-OH for polyubiquitinylation and proteosomal degradation. Similarly, hydroxylation of TRP by PHD and asparaginyl hydroxylase FIH targets TRP-OH for ubiquitinylation and proteosomal degradation contributing to homeostasis. Under hypoxia, HIF-1α translocates to the cell nucleus where it forms heterodimers with the HIF-β subunit and binds to the HRE element (the HRE core binding site sequence is 5'-RCGTG-3' with R being a purine) in the promoter of hypoxia-inducible genes and recruits histone acetyltransferases CREB Binding Protein (CBP)/p300. Hypoxia up-regulates ACE1 which contributes to Ang II production through the RAS pathway. Ang II can contribute to hypoxia through binding to AT1R which initiates signaling events including activation of PKC and c-Src that is required for superoxide production by NADPH oxidases (NOX1 and NOX2). NOX2 also stimulates the production of reactive oxygen species (ROS) by mitochondria. HIF-1α nuclear translocation also activates the transient receptor potential ankyrin 1 (TRPA1) gene expression which leads to increase in intracellular Ca2+ and cell injury. In parallel (not shown), Ang II triggers an increase in cytoplasmic Ca2+ that induces NOX5 to generate H2O2. The Kelch-like erythroid cell-derived protein (KEAP1) is a negative regulator of the nuclear factor (erythroid-derived 2)-like 2 (Nrf2). Under hypoxia, KEAP1 is oxidated by ROS. This leads to the nuclear translocation of phosphorylated Nrf2 which maintains oxidative homeostasis in regulating PHD thereby promoting HIF-1α proteasomal degradation. The Nrf2/MAF (MAF BZIP transcription factor) heterodimers also induce transcription of anti-oxidant-response genes through binding to the antioxidant response element (ARE sequence: 5'-TGACNNNGC-3') and reduce both the concentration of ROS and pro-inflammatory cytokines. In addition, chronic hypoxia triggers HIF-dependent down regulation of the ACE2 gene and activation of ADAM17 which leads to cleavage of the ACE2 protein (not shown).

In humans, three different subunits, HIF-1α, HIF-2α, and HIF-3α have been recognised and several spliced variants of the alpha subunit of HIF have been described (Michel et al., 1999; Semenza, 2000a; Lee et al., 2004). Other members of this family have been described including ARNT2 and ARNT3, which are related to HIF-1β/ARNT1 (Semenza, 2000a). HIF-1α is mainly regulated by a prolyl hydroxylase (PHD) and by an E3 ubiquitin ligase, the von Hippel-Lindau gene product (pVHL). PHD and pVHL trigger HIF-1α degradation at high O_2_ concentration while HIF-1α translocates to the cell nucleus at low O_2_ concentrations and forms heterodimer with the constitutively expressed HIF-1β to activate gene transcription. (Maxwell et al., 1999; Ratcliffe, 2013) (Figure 2). There are four PHD isoforms, three of which are involved in HIF-1α hydroxylation on Pro-402 and Pro-564. HIF activity can also be controlled either through activation of HIF-α transcription or regulation of HIF-α protein synthesis. The former is achieved either through the NF-κB or Jak/Stat3 pathway, while the latter occurs via the PI3K/Akt/mTOR pathway. Both contribute towards cell survival in hypoxic conditions (Brahimi-Horn et al., 2007; Lee et al., 2019). Thus, stabilization of HIF-1α may improve the outcome of COVID-19 by decreasing hypoxia and acting on ACE2 expression (Afsar et al., 2020). PHD and the asparaginyl hydroxylase FIH are also responsible for hydroxylation of TRP leading to their proteasomal degradation (Nagarajan et al., 2017).

The human gene coding for HIF-1α is located at position 14q23.2 on chromosome 14 and consists of 15 exons (size ranging from 85 to 1,321 bp) that are interrupted by 14 introns (Lyer et al., 1998). Five SP1 sites (including three SP1 sites located immediately 5' of the transcription initiation site) and four bHLH-binding E-boxes (starting −141, −243, −254, and −277) are present in the HIF1A promoter. An investigation into HIF-1α gene polymorphism found a dinucleotide repeat in intron 13 (GT14 allele) that was more frequent in Tibetan Sherpas than in the control group composed of Japanese while the GT15 allele was more frequent among Japanese participants (Suzuki et al., 2003). Two non-synonymous polymorphisms in HIF-1α, Pro582Ser [rs11549465] and Ala588Thr [rs11549467], were found to enhance HIF-1α activity (Ollerenshaw et al., 2004). The rs11549465 variant of HIF-1α impacts breast cancer metastasis (Kim et al., 2008) and is associated with COVID-19 susceptibility (Das et al., 2021). No association was found between average altitude and COVID-19 outcomes but there are 34 single nucleotide polymorphisms (SNPs) reported to date for HIF-1α which have not been studied in terms of their association with either average altitude or COVID-19 (Gladek et al., 2017). However, the rs10873142 polymorphism has been associated with a risk of chronic obstructive pulmonary disease (Wang et al., 2018). In addition, genome-wide allelic differentiation comparing Tibetan highlanders with lowland Han demonstrated divergence across 31 SNPs including the most significant SNP (rs4953354). The rs4953354 is located near the *EPAS1* gene encoding HIF-2α which stimulates production of erythrocytes and levels of haemoglobin (Beall et al., 2010; Yi et al., 2010). Epigenetic processes are also essential to the regulation of HIF transcription as illustrated by the CpG methylation of *EPAS1* promoter by DNA methyltransferase 3a which inhibits HIF-2α expression under hypoxic conditions (Lachance et al., 2014).

## The renin-angiotensin system pathway in populations living at high altitude and its imbalance in cases of coronavirus disease 2019

Among the characteristic physiology of high altitude natives, the genes of the renin-angiotensin system (RAS) deserve special attention because they play an important role in the regulation of the pulmonary vascular tone. The search for candidate genes involved in adaptation to hypoxia among 142 young males and females of Quechua origins in Peru, revealed that the I-allele of the angiotensin-converting enzyme (ACE1) gene insertion/deletion (I/D) polymorphism is associated with performance benefits at high altitude (Bigham et al., 2008). The study of pathologies specifically observed with individuals exposed to hypoxic conditions may also highlight abnormal functioning of the RAS pathway. High-altitude pulmonary oedema (HAPE) is a non cardiogenic pulmonary oedema that may develop in otherwise healthy individuals in hypoxic environments. It was reported that significant difference in genotype and allele frequency of the ACE I/D and angiotensinogen (AGT) M235T polymorphism is observed between HAPE patients and HAPE resistant individuals of Indian origin (Stobdan et al., 2011). Of course not all high altitude pathologies (e.g., high-altitude polycythemia) are associated with the RAS pathway genes (Fan et al., 2018).

Short after the first cases of COVID-19, SARS-CoV-2 was identified as the aetiological agent of the disease (Zhou et al., 2020; Zhu et al., 2020), and angiotensin I converting enzyme 2 (ACE2) was demonstrated to be the viral entry receptor for this virus (Qiu et al., 2020; Yan et al., 2020). Before being regarded as coronaviruses receptor, ACE2 was known to play a central role in the RAS, which regulates cardiovascular and renal functions and maintains blood pressure homeostasis as well as fluid and salt balance. Hypertension is a major risk factor for endothelial dysfunction and atherosclerosis. In this regard, uncontrolled high blood pressure values might lead to the development of vascular remodeling and vascular rigidity, which may predispose to heart left ventricular hypertrophy and fibrosis, acute kidney injury, and extensive microthrombosis in coronary and pulmonary circulation (Gallo et al., 2022a). The accumulation of such physiological disorders may severely impact on the prognosis of COVID-19 patients and increase risk of death.

The ACE2 is cell-surface peptidase that converts the vasoconstrictor octapeptide [Asp-Arg-Val-Tyr-Ile-His-Pro-Phe] angiotensin II (Ang II) into the heptapeptide Ang-(1-7) to maintain blood pressure homeostasis. Ang II stimulates the production of E-selectin and plasminogen activator inhibitor-1 (PAI-1), thereby contributing to a prothrombotic state and to atherosclerotic plaque rupture and it also induces the synthesis of aldosterone which activates mineralocorticoid receptors enhancing inflammation, fibrosis, and endothelial damage. In contrast to Ang II, Ang-(1-7) has vasodilator and anti-fibrotic actions. Ang II binds to Ang II type I and type II receptors (AT1R and AT2R) (Wakahara et al., 2007; Arendse et al., 2019). The plasma Ang II levels increase under hypoxic conditions (Zakheim et al., 1976). Carotid body chemoreceptors, which act as the first gate for detecting a rapid change in oxygen tension and composition in the arterial blood, respond to hypoxia by stimulating ventilation activity through the afferent sinus nerve (Leung et al., 2000; Fung et al., 2002; Fung, 2014) and to Ang II through AT1R (Allen, 1998; Fung et al., 2002). Moreover, elevated ACE1 mRNA expression and ACE1 activity in the carotid body are linked to hypoxia (Lam et al., 2004). Both ACE1 and Ang II play roles in hypoxia-induced pulmonary hypertension and vascular remodeling (Morrell et al., 1995; Morrell et al., 2005). Finally, the RAS imbalance determines the clinical outcome of COVID-19 (Devaux et al., 2020; Rysz et al., 2021). Due to the interdependence between the virus and RAS, it should be questioned whether there is a link between hypoxia and the RAS pathway.

## HIF-1α and renin-angiotensin system regulation

**FIGURE 3:**
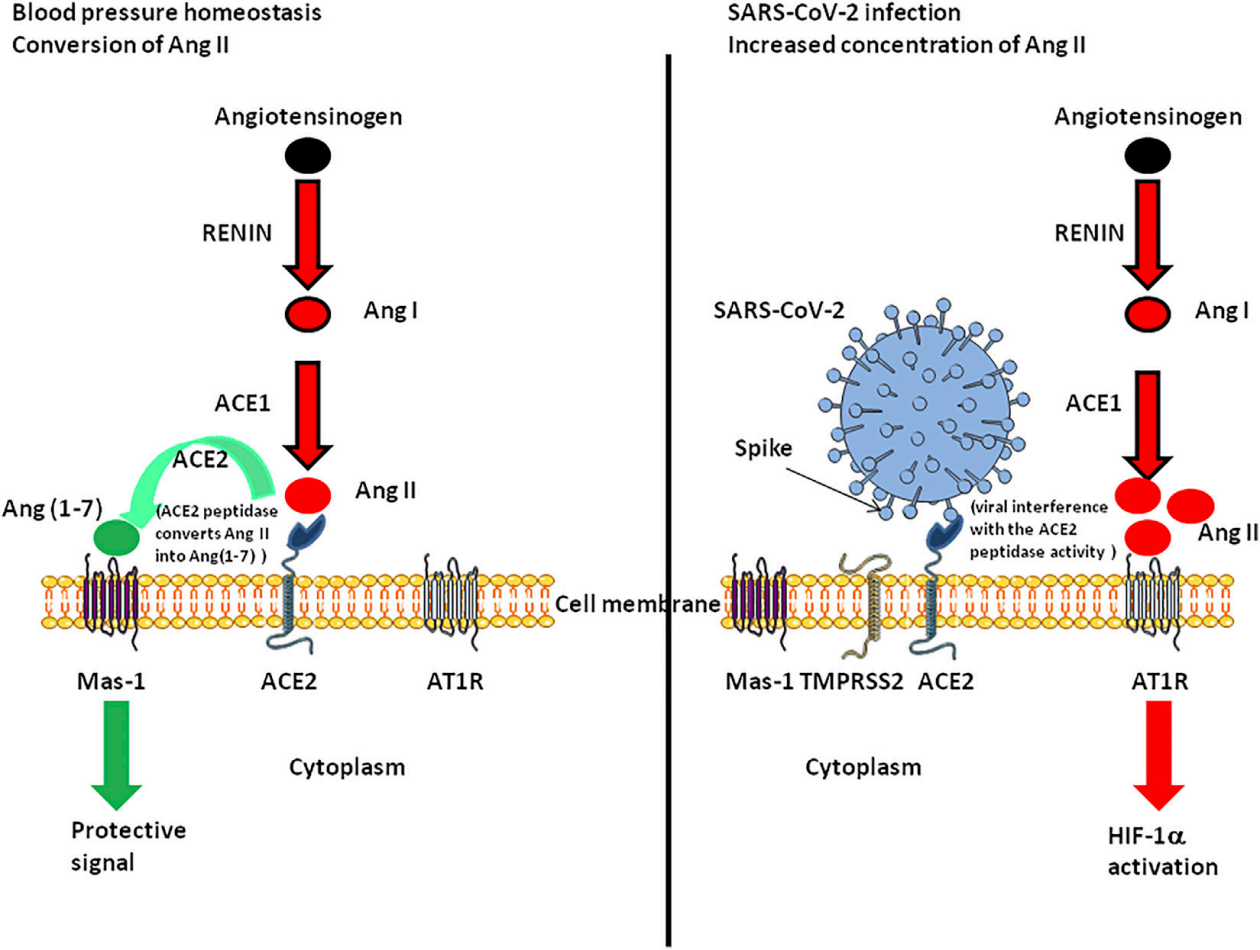
Schematic representation of the functioning of the renin-angiotensin system under normal conditions and during SARS-CoV-2 infection. The left panel illustrates the fact that ACE2 converts Ang II into Ang(1-7). Ang-(1-7) exhibits vasodilatory, anti-proliferative, and anti-inflammatory effects via the G protein-coupled receptor called Mas-1. The right panel illustrates the possible dysfunction of signals when SARS-CoV-2 is attached to its ACE2 receptor. Under this condition Ang(1-7) is no longer synthetised, Ang II accumulates and binds AT1R, leading to HIF-1α induction.

One aspect of high-altitude hypoxia that is relevant to COVID-19 is variations in ACE2 tissue expression and Ang II plasma concentration. Ang II contributes to hypoxia through AT1R or AT2R signaling (Munzenmaier and Greene, 1996; Wolf et al., 2004). In a rat model, AT1R expression was doubled after 4 weeks of chronic hypoxia, leading to enhanced carotid body sensitivity to Ang II (Leung et al., 2000). Another study demonstrated that foetal hypoxia increases cardiac AT2R expression (Xue et al., 2011). In healthy humans exposed to isocapnic intermittent hypoxia, a significant increase in SaO_2_ was observed which could be abolished by treatment with Losartan, suggesting a role for AT1R (Foster et al., 2010). Interestingly, we previously reported that COVID-19 patients have increased concentrations of plasma Ang II and modulation of ACE2 (Osman et al., 2021). The higher expression of Ang II should lead to worsening hypoxia (Figure 3). Transcription of ACE1 mRNA is up-regulated in pulmonary artery smooth muscle cells during hypoxia along with activation of HIF-1α, while ACE2 mRNA increased during the early stage of hypoxia only (Zhang et al., 2009). However, this conclusion remains the subject of debate (Hampl et al., 2015; Oarhe et al., 2015; Joshi et al., 2019). Moreover, HIF-1α was found to be activated in alveolar type 2 cells (target for SARS-CoV-2), during acute lung injuries (McClendon et al., 2017) while Ang II-induced renal injury requires activation of HIF-1α (Zhu et al., 2011).

It has been speculated that hypoxia-induced HIF-1α upregulates the metalloproteinase ADAM17, which can cleave ACE2 (Serebrovska et al., 2020). More recently, it has been demonstrated that hypoxia, as well as treatment with a PHD inhibitor reduces ACE2 expression and inhibits SARS-CoV-2 entry in the human lung epithelial cells via an HIF-1α dependent pathway (Wing et al., 2021). Hypoxia was found to decrease the cell surface levels of heparan sulfate required for SARS-CoV-2 attachment by reducing the expression of syndecan-1 and syndecan-3 (Prieto-Fernandez et al., 2021). Although ethnic polymorphisms in ACE2 are documented (Benetti et al., 2020; Cao et al., 2020; Othman et al., 2020; Suryamohan et al., 2021), it is not known whether there are specific variants to people living at high altitude. The same hold for ACE1 polymorphism (Marshall et al., 2002; Harrap et al., 2003; Gupta et al., 2009; Yamamoto et al., 2021).

## Hypoxia-inducible factor and vascular inflammation

**FIGURE 4:**
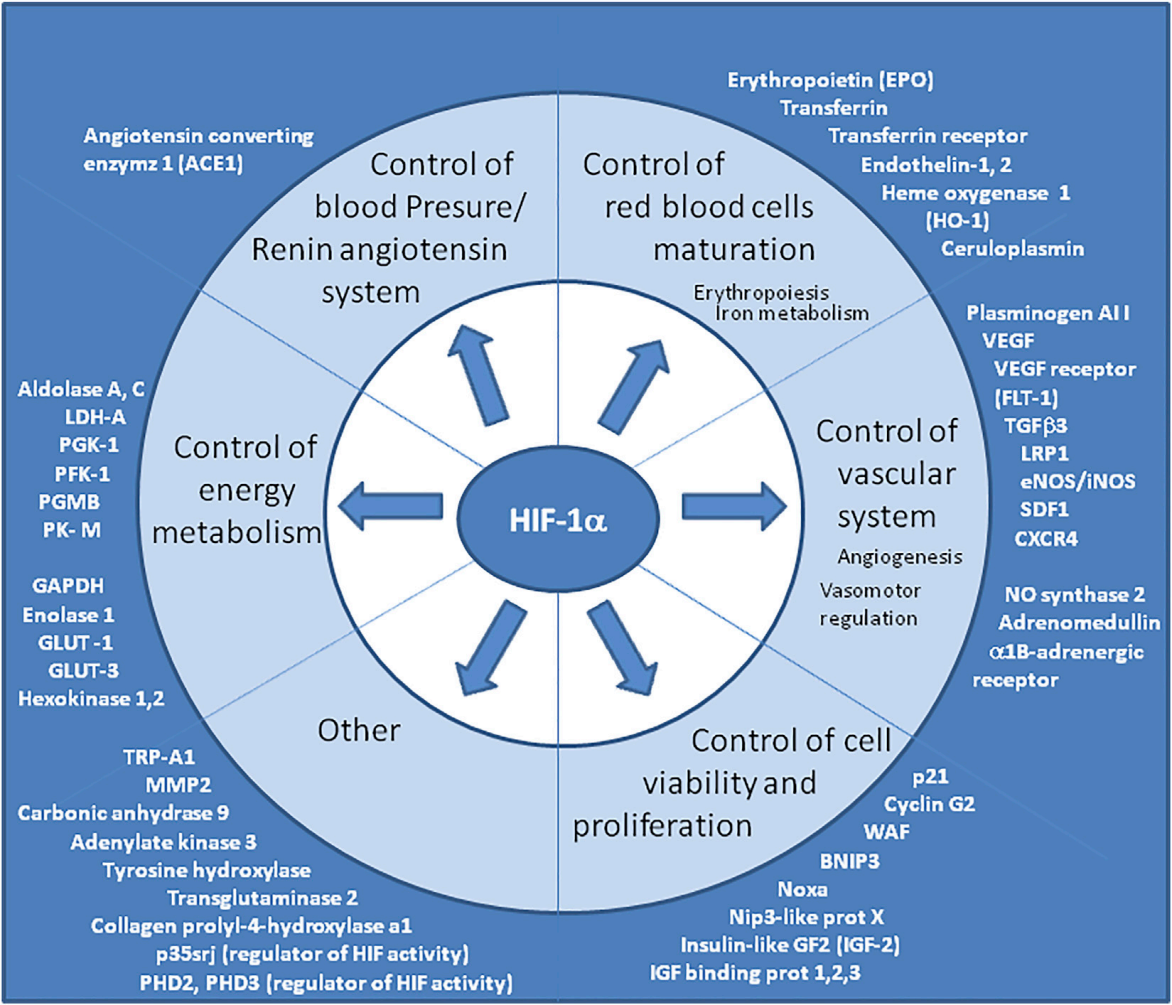
Genes upregulated by HIF “master regulator”. The consensus DNA sequence for HIF-1α/HIF-1β binding is common for many genes upregulated during hypoxia. Representative list of genes upregulated by HIF, however this list is not exhaustive and grows continuously. It is worth noting that several genes involved in the renin-angiotensin system, in the development and functioning of the vascular system (which modulate vascular tone or promote angiogenesis) and in erythropoiesis, belong to this list. HIF-1α is also known to upregulate TRP-A1 and p35srj. The p35srj protein is an alternatively spliced isoform of MRG1 which inhibits HIF-1 transactivation by blocking the HIF-1α/p300 interaction.

Ang II induces HIF-1α which activates the expression of vasoconstrictor endothelin-1 in vascular endothelial cells (Semenza, 2000b). HIF-1α also controls the expression of erythropoietin which regulates blood oxygen (Maxwell et al., 1993). Finally, HIF-1α regulates the expression of dozen of genes (Figure 4), including heme oxygenase-1, angiogenic factor vascular endothelial growth factor (VEGF), and VEGF receptor FLT-1. (Crews, 1998; Semenza, 2000b; Lee et al., 2004; Gaber et al., 2005). HIF-1α induced expression of the VEGF gene requires the histone acetyl transferase coactivators p300 and p160 steroid receptor coactivator 3 (SRC-3) (Wang et al., 2010). The B-cell lymphoma (bcl)-2 protein also regulates HIF-1α-mediated expression of VEGF in hypoxic conditions by acting on HIF-1α stabilization through a mechanism that involves the BH4 domain of bcl-2 (Trisciuoglio et al., 2011). The HIF-1 binding site is located at approximately -1 kbp in the VEGF promoter and a drug such as the tyrosine kinase inhibitor gefitinib decreases VEGF mRNA expression by down-regulating HIF-1α expression (Pore et al., 2006). VEGF was reported to be activated through HIF-1α after viral infection (Ripoli et al., 2010; McFarlane et al., 2011), leading to increase in vascular permeability (Colgan et al., 2020). Vascular permeability allows the extravasation of plasma proteins that form a primitive scaffold for migrating endothelial cells (Zimna and Kurpisz, 2015). Endothelial dysfunction may participate to the increased myogenic tone of resistance arteries through the activation of the RAS, endothelin-1, catecholamines, and growth factors production, leading to vasoconstriction, increase of blood pressure, vascular remodeling and then to a risk of artherosclerosis (Gallo et al., 2022b). Since vascular leakage could lead to septic shock, several HIF-1α-regulated genes have been associated with the protection of vascular barrier function during hypoxia, (Mahabeleshwar et al., 2012) thereby counterbalancing the adverse effects of VEGF.

In addition, AT1R blocking decreases the VEGF expression induced by Ang II in peripheral blood mononuclear cells (Takahashi et al., 2005). Patients with COVID-19 often develop shortness of breath and dyspnoea which triggers local hypoxia and induces HIF-1α activation and VEGF production (Cao, 2021). Hypoxia is associated with an up-regulation in VEGF mRNA expression while ACE2 mRNA expression is upregulated under 2% hypoxia at 6 hours and down-regulated at 48 h (Imperio et al., 2021). Thus, hypoxia acutely enhances ACE2 but chronically reduces ACE2 and TMPRSS2, and this should lead to decreased susceptibility to SARS-CoV-2 in people living in conditions where oxygen is limited. Ang II behaves as a mediator of oxidative stress, in which reactive oxygen species (ROS) are important signaling intermediates in several signal transduction pathways such as vascular remodeling (Duprez, 2006). Vascular inflammation induced by Ang II is mainly mediated by AT1R associated with an increased production of ROS via the NADPH oxidase (Griendling et al., 1994; Rajagopalan et al., 1996; Dandona et al., 2003).

## Hypoxia-inducible factor regulates antiviral and pro-inflammatory responses

Beside regulating ACE2 expression and RAS, HIF-1α has also been reported to regulate type 1 interferon (IFN) antiviral response and the production of cytokines (Nizet and Johnson, 2009; Jahani et al., 2020). The innate immune response against viruses is triggered by the recognition of pathogen-associated molecular patterns through different receptors including Toll-like receptors (TLRs) which are proteins on the cell membrane or within endosomes, nucleotide-binding and oligomerization domain (NOD)-like receptors which are cytoplasmic sensors, and retinoic acid inducible gene I (RIG-I) which are cytosolic helicases (Chen et al., 2009). Each TLR has its own specificity (Lester and Li, 2014). TLR3 sense single stranded RNA (ssRNA) viruses, double stranded RNA (dsRNA) viruses, and DNA viruses. SARS-CoV-2, a positive-sense RNA virus, can be sensed both as ssRNA and as dsRNA (produced during its replication), leading to the activation of IFN regulatory factor (IRFs). The activation of IRFs results in homo-, or hetero-dimerization of IRF-3 and/or IRF-7 and their subsequent translocation to the nucleus where they bind to IFN promoters. Under normoxic conditions the alarmin HMGB1 activates IRF-5, IRF-3 and NF-κB in monocytes leading to expression of IFN and inflammatory cytokines but, surprisingly, under hypoxic conditions monocytes fail to produce IFN through repression of IFR-5 by HIF-1 α (Santos and Andrade, 2017; Peng et al., 2021). At low O_2_ concentration, the HIF-1α increase promotes cell phagocytic activity and stimulates pro-inflammatory cytokines synthesis (Colgan et al., 2020). The role of HIF-1α in the induction of cytokines production at the site of inflammation was previously reported for patients with severe H1N1 viral pneumonia (Guo et al., 2017). Similarly, severe COVID-19 is characterized by lung injury as result of high levels of inflammatory cytokines (Fan et al., 2018; Huang et al., 2020; Boumaza et al., 2021). Upon SARS-CoV-2 infection, the viral ORF3a induces mitochondrial damage and the generation of oxygen species activates HIF-1α enhancing SARS-CoV-2 infection and inflammatory responses (Tian et al., 2021).

## Anti-SARS-CoV-2 properties of curcumin through anti-HIF-1α function

Ethnobotanical studies reported that food and spices have a symbolic value among the hinduistically-influenced ethnic groups of Nepal and they constitute an integral part of traditional medicine. The perennial herb *Curcuma longa* belonging to Zingiberaceae, is an important spice in *Dal*, the most frequently eaten dish of lentils in rural Nepal. Tumeric (an orange-yellow crystalline powder insoluble in water), is also used in the composition of *Causa Limena*, a traditional Peruvian dish. Beside its used as a dietary spice, the active ingredient of the turmeric rhizome, curcumin (diferuloylmethane) (Lin, 2007; Zhou et al., 2011), is also marketed for its anti-inflammatory properties. Moreover, *Curcuma longa* is known to be used in Nepal in medicinal prescriptions for ‘purification of the blood (Eigner and Schilz, 1999). It has been established that high altitude associated chronic hypobaric hypoxia induces skeletal muscle atrophy and that curcumin has muscle sparing effects and enhances muscle mass (Chaudhary et al., 2018), which can also encourage local consumption of tumeric. This raise questions about whether the curcumin-rich diet of people living at high altitudes may influence their susceptibility to COVID-19.

Numerous compounds derived from medicinal plants have been investigated in the search for antiviral action against SARS-CoV-2. Curcumin has been described as having antiviral, antiplatelet, antioxidant, antibacterial, and anti-inflammatory properties which could be beneficial to COVID-19 patients (Paciello et al., 2020; Rattis et al., 2021). In a model of acute lung injury, treatment with curcumin was reported to increase PaO_2_, to decrease pulmonary oedema and to improve lung function (Cheng et al., 2018). At the molecular level, among other antiviral mechanisms (Rattis et al., 2021), curcumin has been reported to show high affinity for both SARS-CoV-2 spike (S) glycoprotein and its cellular receptor, ACE2 and to bind TMPRSS2, suggesting a capacity to block viral attachment to target cells (Maurya et al., 2020; Motohashi et al., 2020; Shanmugarajan et al., 2020). Curcumin is also thought to interfere with endosomal acidification, an essential step in the SARS-CoV-2 replication cycle (Vishvakarma et al., 2011). Moreover, curcumin is thought to inhibit ACE1 activity (Abd Allah and Gomaa, 2015), thereby preventing the production of Ang II. Interestingly clinical studies reported reduction of fever, cough, and dyspnea in COVID-19 patients receiving nanoencapsulated curcumin (Hassaniazad et al., 2020; Tahmasebi et al., 2020; Valizadeh et al., 2020; Ahmadi et al., 2021; Pawar et al., 2021; Saber‐Moghaddam et al., 2021; Trigo‐Gutierrez et al., 2021). At the onset of the COVID-19 pandemia, the French agency for health safety (ANSES) warned about using curcumin during COVID-19 due to the potential risks of reducing the immune defenses in an infectious context (referral number 2020-SA-0045 published on 10 April, 2020). In addition warning letters from the U.S. Food and Drug Administration (FDA) were published with regards to the use of curcumin-containing products for COVID-19 treatment (https://www.fda.gov/inspections-compliance-enforcement-and-criminal-investigations/compliance-actions-and-activities/warning-letters). However, curcumin is largely used in countries such as India without any noticeable side effects. Pharmacokinetic data has shown that curcumin undergoes rapid metabolism with glucuronidation and sulfation in the liver and excretion in the feces, which leads to its poor systemic bioavailability. Several synthetic curcumin analogs have been designed to overcome these drawbacks and gain efficiency while reducing toxicity (Vyas et al., 2013; Chainoglou and Hadjipavlou-Litina, 2019; Shimizu et al., 2020).

**FIGURE 5:**
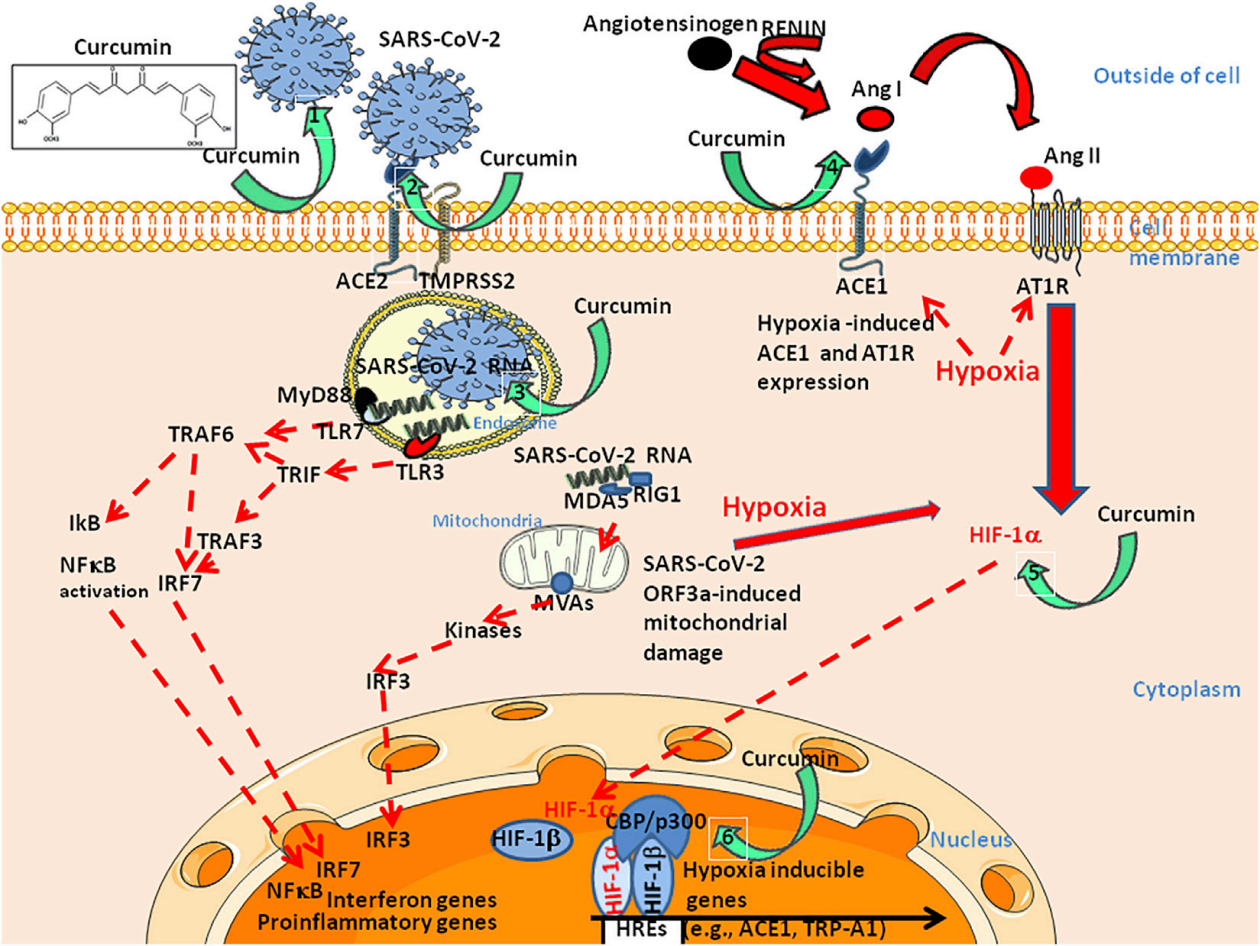
Schematic diagram illustrating six of the potential curcumin targets during SARS-CoV-2 infection of susceptible cells with a specific focus on: Left: viral entry pathway (right panel, targets 1–3) and, Right: Ang II induced hypoxia pathway (left panel, targets 4–6). Regarding the viral entry pathway, curcumin prevents the binding of the SARS-CoV-2 S protein to the ACE2 receptor by binding to both proteins (1,2). In addition, curcumin prevents endosomal acidification required for the initiation of viral replication (3). Regarding the Ang II pathway, curcumin inhibit ACE1 (4), thus preventing the accumulation of Ang II. Moreover, curcumin prevents hypoxia-induced HIF1-α upregulation (5) and p300 acetyl transferase activity within the HIF1-α/β/CBP-p300 complex (6). Upper left, chemical structure of curcumin (diferuloylmethane). Effects of curcumin on Trp ion channels such as TrpA1 and TrpV1, have also been reported (not shown). ACE: Angiotensin converting enzyme; Ang: Angiotensin; AT1R: Ang II type I receptor; CBP/p300: Histone acetyltransferases CREB Binding Protein (CBP)/p300; IRF: Interferon regulatory factor; MDA: Melanoma differentiation-associated gene; MVAs: Mitochondrial antiviral-signal protein; RIG: Retinoic acid inducible gene; TMPRSS2: Transmembrane serine protease two; TLR: Toll like receptor; TRAF: Tumour necrosis factor receptor-associated factor; TRIF: TIR-domain-containing adapter-inducing interferon.

In addition, it is interesting to note that a growing body of evidence indicates that curcumin prevents hypoxia-induced upregulation of HIF-1α (Bae et al., 2006; Bahrami et al., 2018; Ouyang et al., 2019; Moulin et al., 2020; Sarighieh et al., 2020). Finally, curcumin is considered a natural p300 histone acetyltransferase inhibitor (Sunagawa et al., 2011), likely inhibiting the functional transcription complex HIF-1/CBP-p300 (Figure 5). Deepening the molecular mechanism of action of curcumin on HIF could promote the development of effective candidates for SARS-CoV-2 therapy. Preventing transactivation of HIF-1α by peptide inhibitors of HIF-1/CBP-p300 interaction (previously shown to suppress tumor growth) (Kung et al., 2001; Qin et al., 2021) or other compounds with similar effects (Freedman et al., 2002; Jayatunga et al., 2015), could be interesting to test in order to evaluate their possible benefit in the treatment of COVID-19. Alternatively, Bousquet and others have recently proposed that fermented vegetables and spices such as curcumin act as activators of the nuclear transcription factor erythroid-derived 2 like 2 factor (Nrf2) and Transient Receptor Potential Ankyrin 1 and Vanillin 1 (TRPA1-V1) channels and may help as complementary treatments in the control of COVID-19 symptoms (such as cough and respiratory symptoms) (Bousquet et al., 2022). Because patients experienced a very rapid (less than 5 min) improvement of some of the symptoms after taking curcumin, Bousquet and others hypothesized that the mechanism of action of this natural compound could be a transient desensitization of TRPA1-V1 channels (Bousquet et al., 2021).

## Negative feedback loop on HIF-1α

Under normoxic conditions the cytoplasmic form of Nrf2 (a 605 amino acids protein) is associated with the Kelch-like ECH-associated protein 1 (Keap1). Under hypoxic conditions and upon excessive ROS production, oxidation of several cysteines on Keap1 interrupts Nrf2-Keap1 interaction and leads to Nrf2 nuclear translocation. In the nucleus, Nrf2 forms heterodimers with small MAF (sMAF) proteins and binds to regulatory antioxidant response element (ARE), and CBP/p300 through interaction with the NEH4 and NEH5 domain thereby inducing the transcription of plethora of genes. Among these genes several encode products playing important role in the survival of cell such as antioxidant genes including thioredoxin (TRX1), peroxiredoxin-1 (PRDX1), sulfiredoxin-1 (SRXN1), glutathione peroxidase (GPx), glutathione S transferase (GST), superoxide dismutase (SOD), heme oxygenase-1 (HO-1), or detoxification enzymes such as NADPH quinone oxidoreductase 1 (NQO1), and ferritin. The coordinate action of Nrf2 target genes bring back the cell to redox homeostasis (Kobayashi et al., 2016; Jeddi et al., 2017; Toh and Warfel, 2017). It has been assumed that ROS are produced by an NADPH oxidase that reduces O2 to superoxide anion which is subsequently converted to hydrogen peroxide (H_2_O_2_) by SOD triggering the reduction of ROS levels leading to HIF-1 modulation. Nrf2 also binds to an ARE sequence located approximately 30 kilobases upstream of *HIF1A* upregulating the production of HIF-1α (Lacher et al., 2018). An alternative pathway of HIF-1α activation by Nrf2 is likely through TRX1 (Hawkins et al., 2016). However, under specific conditions Nrf2 levels can also be decreased during hypoxia leading to decreased activity of HIF-1α (Potteti et al., 2021). Nrf2 also regulates the coordinated expression of cytoprotective genes, including HO-1 which protects cells from oxidative stress (Deshmukh et al., 2017). In contrast, HO-1 was also found to play an important role in mediating the proangiogenic effects of stromal cell-derived factor 1 (SDF-1) through CXCR4 7-transmembrane receptor signalling (Deshane et al., 2007). Finally, Nrf2 blocks the transcription of proinflammatory cytokines (Kobayashi et al., 2016). Nrf2 was reported to auto-regulate its expression by binding to ARE-like elements present in the proximal region of the Nrf2 promoter and to be negatively regulated by BACH1 that dimerizes with sMAF (Shaw and Chattopadhyay, 2019).

In addition, HIF-1α was reported to activate the transcription of p35srj. This protein, also called CBP/p300 interacting transactivator with an ED-rich tail 2 (CITED2), is an alternatively spliced isoform of the melanocyte-spepcific gene related gene 1 (MRG1) chromodomain-containing protein which inhibits HIF-1 transactivation by blocking the HIF-1α/p300 interaction (Bhattacharya et al., 1999). CITED2 is induced during hypoxia and is competing with NAD of HIF-1α for binding to the CH1 domain of p300 (Yoon et al., 2011). Moreover, the prolyl hydrolase PHD2 is upregulated by hypoxia and its downstream promoter contains a functional hypoxia-responsive cis-regulatory element HRE, thus limiting HIF-1α signaling (Metzen et al., 2005; Fong and Takeda, 2008). Finally, in addition to inducing hyperacetylation of histones by targeting histone deacetylases (HDACs), histone deacetylase inhibitors (HDAIs) such as geldanamycin or apicidin have been found to be potent inhibitors of HIF function possibly through the HIF-1α ligand, Hsp90, or through acetylation of the HIF-CAD domain (aa 786-826) (Liang et al., 2006; Kim et al., 2007).

## Discussion

Large variations in susceptibility to SARS-CoV-2 infection and severity of COVID-19 across the world may have multiple underlying causes, including environmental and genetic factors (Frutos et al., 2020; Gautret et al., 2020; Haug et al., 2020; Ioannidis, 2020; Fricke-Galindo and Falfan-Valencia, 2021). The question was recently asked as to whether living in high-altitude regions might protect against COVID-19 (Arias-Reyes et al., 2020; Pun et al., 2020). By critically examining the influence of chronic hypoxia on susceptibility to and severity of COVID-19, the question can be redirected as to the role that the HIF-1α molecule plays on SARS-CoV-2.

Molecular arguments have been accumulated *in vitro* suggesting that chronic hypoxia could modulate the genes involved in cell susceptibility to SARS-CoV-2, such as ACE2. Under chronic hypoxia ACE1 expression is upregulated by HIF-1α in human pulmonary artery smooth muscle cells while ACE2 expression is markedly decreased (Osman et al., 2021). HIF-1α was also reported to be able to inhibit the SARS-CoV-2 infection of lung epithelial cells by reducing ACE2 expression (Prieto-Fernandez et al., 2021; Wing et al., 2021). This leads to the hypothesis that hypoxia could have a beneficial effect on the molecular crosstalk that regulates the severity of COVID-19 through HIF-1α. Moreover, an association of rs11549465 variant of HIF-1α with COVID-19 susceptibility has been reported (Das et al., 2021). If hypoxia reduces the capacity of SARS-CoV-2 to attach and enter target cells, in contrast activation of HIF-1α may reduce antiviral immune defenses and exacerbate pro-inflammatory responses and thrombosis which are detrimental to people infected with SARS-CoV-2. The results reported in the literature are dependent on the duration of hypoxia, the cell type selected for *in vitro* experiment, and the tissues and organs (e.g., lungs, kidneys) chosen. This highlights the fact that gene regulation by HIF-1α is a complex patchwork of signaling pathways with sometimes opposite effects.

Although fully adapted for signaling pathway characterization, the *in vitro* cellular model should be used with caution when extrapolating to biological processes in the context of an organ or an individual. In humans, SARS-CoV-2 pathogenesis start with the interaction of the viral spike with ACE2 and TMPRSS2 on the surface of type II alveolar cells (lung). Following infection, a functional defect in ACE2 leads to the accumulation of Ang II. Interestingly, for ACE1 which catalyses the proteolysis of Ang I to form Ang II, an ACE1 47-bp insertion/deletion (I/D) polymorphism has been associated with SaO_2_ among Peruvian, with I/I allele individuals showing higher SaO_2_ compared to I/D and D/D individuals (Bigham et al., 2008; Simonson et al., 2010). Ang II-induced hypoxia activates HIF-1α in macrophages triggering IFR5 and IFR3 repression and the reduction of IFN. Activation of HIF-1α in epithelial cells aggravates the defect in the ACE2 production. At the same time, HIF-1α activation leads to overproduction of proinflammatory cytokines and a VEGF-induced vascular leakage in endothelial capillary cells. In the kidneys, hypoxia leads to cell apoptosis, fibrosis of the kidney and loss of peritubular capillaries (kidneys) (Nangaku and Fujita, 2008). Inhibition of PHD by hypoxia downregulates the vascular expression of the Ang II receptor highlighting the existence of feedback loop of regulation (Matsuura et al., 2011). Interestingly, the *EGLN1* gene (PHD2) shows evidence of positive selection in both Tibetans and Andeans of rs186996510 and rs12097901alleles (Bigham et al., 2010; Bhandari et al., 2017). Moreover, in preclinical chronic mountain sickness, an hypermethylation of *EGLN1* has been reported to diminish PHD2 expression and thereby enable transcription of HIF-1α (Julian, 2017). In contrast, a NO synthase three G894T polymorphism is associated with the lower expression of HIF-1α (Armenis et al., 2021). Hypoxia also induces Col-I mRNA expression and collagen synthesis in human lung fibroblasts which can be blocked by AT1R and AT2R inhibitors (Liu et al., 2013).

Until further investigations take place, data regarding the lower incidence and/or morbidity of SARS-CoV-2 in populations living at high altitudes must be considered speculative. However, hypoxia could be beneficial in reducing virus binding and entry into cells, while being detrimental to the outcome of patients once infected with SARS-CoV-2. These different issues appear to be orchestrated through HIF-1α regulation. A better understanding of the mechanisms of HIF-1α stabilization/degradation and molecular crosstalk between HIF-1α and other cellular proteins will likely suggest new targets and new therapeutic strategies that may help to overcome COVID-19.

## Data Availability

The original contributions presented in the study are included in the article/Supplementary Materials, further inquiries can be directed to the corresponding author.

## References

[B1] Abd Allah E. S. H. Gomaa A. M. S. (2015). Effects of curcumin and captopril on the functions of kidney and nerve in streptozotocin-induced diabetic rats: Role of angiotensin converting enzyme 1. Appl. Physiol. Nut. Metab. 40 (10), 1061–1067. 10.1139/apnm-2015-0145 26398443

[B2] Afsar B. Kanbay M. Elsurer Afsar R. (2020). Hypoxia inducible factor-1 protects against COVID-19: A hypothesis. Med. Hypotheses 143, 109857. 10.1016/j.mehy.2020.109857 32464493 PMC7238987

[B3] Ahmadi R. Salari S. Sharifi M. D. Reihani H. Rostamiani M. B. Behmadi M. (2021). Oral nano‐curcumin formulation efficacy in the management of mild to moderate outpatient COVID‐ 19: A randomized triple‐blind placebo‐controlled clinical trial. Food Sci. Nutr. 9 (8), 4068–4075. 10.1002/fsn3.2226 34401058 PMC8358331

[B4] Allen A. M. (1998). Angiotensin AT1 receptor-mediated excitation of rat carotid body chemoreceptor afferent activity. J. Physiol. 510, 773–781. 10.1111/j.1469-7793.1998.773bj.x 9660892 PMC2231066

[B5] Arendse L. B. Danser A. H. J. Poglitsch M. Touyz R. M. Burnett J. C. Llorens-Cortes C. (2019). Novel therapeutic approaches targeting the Renin-Angiotensin System and associated peptides in hypertension and heart failure. Pharmacol. Rev. 71, 539–570. 10.1124/pr.118.017129 31537750 PMC6782023

[B6] Arias-Reyes C. Zubieta-DeUrioste N. Poma-Machicao L. Aliaga-Raduan F. Carvajal-Rodriguez F. Dutschmann M. (2020). Does the pathogenesis of SARS-CoV-2 virus decrease at high-altitude? Respir. Physiol. Neurobiol. 277, 103443. 10.1016/j.resp.2020.103443 32333993 PMC7175867

[B7] Armenis I. Kalotychou V. Tzanetea R. Konstantopoulos K. Rombos I. (2021). The effect of endothelial nitric oxide synthase G894T and T786C polymorphisms on hypoxia-inducible factor-1 alpha expression in sickle cell disease. Nitric Oxide. 111-112, 31–36. 10.1016/j.niox.2021.03.004 33812003

[B8] Bae M. K. Kim S. H. Jeong J. W. Lee Y. M. Kim H. S. Kim S. R. (2006). Curcumin inhibits hypoxia-induced angiogenesis via down-regulation of HIF-1. Oncol. Rep. 15, 1557–1562. 10.3892/or.15.6.1557 16685395

[B9] Bahrami A. Atkin S. L. Muhammed Majeed M. Amirhossein Sahebkar A. (2018). Effects of curcumin on hypoxia-inducible factor as a new therapeutic target. Pharmacol. Res. 137, 159–169. 10.1016/j.phrs.2018.10.009 30315965

[B10] Bärtsch P. Gibbs J. S. R. (2007). Effect of altitude on the heart and the lungs. Circulation 116, 2191–2202. 10.1161/CIRCULATIONAHA.106.650796 17984389

[B11] Beall C. M. Cavalleri G. L. Deng L. Elston R. C. Gao Y. Knight J. (2010). Natural selection on EPAS1 (HIF2alpha) associated with low hemoglobin concentration in Tibetan highlanders. Proc. Natl. Acad. Sci. U. S. A. 107, 11459–11464. 10.1073/pnas.1002443107 20534544 PMC2895075

[B12] Beall C. M. (2003). High-altitude adaptations. Lancet 362, s14–s15. 10.1016/S0140-6736(03)15058-1 14698112

[B13] Beall C. M. Laskowski D. Erzurum S. C. (2012). Nitric oxide in adaptation to altitude. Free Radic. Biol. Med. 52 (7), 1123–1134. 10.1016/j.freeradbiomed.2011.12.028 22300645 PMC3295887

[B14] Beall C. M. (2007). Two routes to functional adaptation: Tibetan and andean high-altitude natives. Proc. Natl. Acad. Sci. U. S. A. 104, 8655–8660. 10.1073/pnas.0701985104 17494744 PMC1876443

[B15] Benetti E. Tita R. Spiga O. Ciolfi A. Birolo G. Bruselles A. (2020). *ACE2* gene variants may underlie interindividual variability and susceptibility to COVID-19 in the Italian population. Eur. J. Hum. Genet. 28, 1602–1614. 10.1038/s41431-020-0691-z 32681121 PMC7366459

[B16] Bhandari S. Zhang X. Cui C. Yangla Liu L. Ouzhuluobu (2017). Sherpas share genetic variations with Tibetans for high-altitude adaptation. Mol. Genet. Genomic Med. 5 (1), 76–84. 10.1002/mgg3.264 28116332 PMC5241213

[B17] Bhattacharya S. Michels C. L. Leung M. K. Arany Z. P. Kung A. L. Livingston D. M. (1999). Functional role of p35srj, a novel p300/CBP binding protein, during transactivation by HIF-1. Genes. Dev. 13 (1), 64–75. 10.1101/gad.13.1.64 9887100 PMC316375

[B18] Bigham A. Bauchet M. Pinto D. Mao X. Akey J. M. Mei R. (2010). Identifying signatures of natural selection in Tibetan and andean populations using dense genome scan data. PLoS Genet. 6 (9), e1001116. 10.1371/journal.pgen.1001116 20838600 PMC2936536

[B19] Bigham A. W. (2016). Genetics of human origin and evolution: High-altitude adaptations. Curr. Opin. Genet. Dev. 41, 8–13. 10.1016/j.gde.2016.06.018 27501156 PMC5161537

[B20] Bigham A. W. Kiyamu M. Leon-Velarde F. Parra E. J. Rivera-Ch M. Shriver M. D. (2008). Angiotensin-converting enzyme genotype and arterial oxygen saturation at high altitude in Peruvian Quechua. High. Alt. Med. Biol. 9, 167–178. 10.1089/ham.2007.1066 18578648 PMC3140306

[B21] Boumaza A. Gay L. Mezouar S. Bestion E. Diallo A. B. Michel M. (2021). Monocytes and macrophages, targets of severe acute respiratory syndrome coronavirus 2: The clue for coronavirus disease 2019 immunoparalysis. J. Infect. Dis. 224 (3), 395–406. 10.1093/infdis/jiab044 33493287 PMC7928817

[B22] Bousquet J. Czarlewski W. Zuberbier T. Mullol J. Blain H. Cristol J. P. (2021). Potential interplay between Nrf2, TRPA1, and TRPV1 in nutrients for the control of COVID-19. Int. Arch. Allergy Immunol. 182 (4), 324–338. 10.1159/000514204 33567446 PMC8018185

[B23] Bousquet J. Haahtela T. Blain H. Czarlewski W. Zuberbier T. Bedbrook A. (2022). Available and affordable complementary treatments for COVID‐19: From hypothesis to pilot studies and the need for implementation. Clin. Transl. Allergy 12 (3), e12127. 10.1002/clt2.12127 35344297 PMC8967265

[B24] Brahimi-Horn M. C. Chiche J. Pouysségur J. (2007). Hypoxia and cancer. J. Mol. Med. 85 (12), 1301–1307. 10.1007/s00109-007-0281-3 18026916

[B25] Brouqui P. Amrane S. Million M. Cortaredona S. Parola P. Lagier J. C. (2021). Asymptomatic hypoxia in COVID-19 is associated with poor outcome. Int. J. Infect. Dis. 102, 233–238. 10.1016/j.ijid.2020.10.067 33130200 PMC7604151

[B26] Cao Y. Li L. Feng Z. Wan S. Huang P. Sun X. (2020). Comparative genetic analysis of the novel coronavirus (2019-nCoV/SARS-CoV-2) receptor ACE2 in different populations. Cell. Discov. 6, 4–7. 10.1038/s41421-020-0147-1 32133153 PMC7040011

[B27] Cao Y. (2021). The impact of the hypoxia-VEGF-vascular permeability on COVID-19-infected patients. Exploration 1, 20210051. 10.1002/EXP.20210051 35434726 PMC8653011

[B28] Chainoglou E. Hadjipavlou-Litina D. (2019). Curcumin analogues and derivatives with anti-proliferative and anti- inflammatory activity: Structural characteristics and molecular targets. Expert Opin. Drug Discov. 14 (8), 821–842. 10.1080/17460441.2019.1614560 31094233

[B29] Chaudhary P. Sharma Y. K. Sharma S. Singh S. N. Suryakumar G. (2018). High altitude mediated skeletal muscle atrophy: Protective role of curcumin. Biochimie 156, 138–147. 10.1016/j.biochi.2018.10.012 30347230

[B30] Chen G. Shaw M. H. Kim Y. G. Nunez G. (2009). NOD-Like receptors: Role in innate immunity and inflammatory disease. Annu. Rev. Pathol. 4, 365–398. 10.1146/annurev.pathol.4.110807.092239 18928408

[B31] Cheng K. Yang A. Hu X. Zhu D. Liu K. (2018). Curcumin attenuates pulmonary inflammation in lipopolysaccharide induced acute lung injury in neonatal rat model by activating peroxisome proliferator-activated receptor γ (PPARγ) pathway. Med. Sci. Monit. 24, 1178–1184. 10.12659/MSM.908714 29480285 PMC5839073

[B32] Colgan S. P. Furuta G. T. Taylor C. T. (2020). Hypoxia and innate immunity: Keeping up with the HIFsters. Annu. Rev. Immunol. 38, 341–363. 10.1146/annurev-immunol-100819-121537 31961750 PMC7924528

[B33] Couzin-Frankel J. (2020). The mystery of the pandemic's 'happy hypoxia. Science 368, 455–456. 10.1126/science.368.6490.455 32355007

[B34] Crews S. T. (1998). Control of cell lineage-specific development and transcription by bHLH–PAS proteins. Genes. Dev. 12, 607–620. 10.1101/gad.12.5.607 9499397

[B35] Dames S. A. Martinez-Yamout M. de Guzman R. N. Dyson H. J. Wright P. E. (2002). Structural basis for Hif-1α/CBP recognition in the cellular hypoxic response. Proc. Natl. Acad. Sci. U. S. A. 99 (8), 5271–5276. 10.1073/pnas.082121399 11959977 PMC122759

[B36] Dandona P. Kumar V. Aljada A. Ghanim H. Syed T. Hofmayer D. (2003). Angiotensin II receptor blocker valsartan suppresses reactive oxygen species generation in leukocytes, nuclear factor-kappa B, in mononuclear cells of normal subjects: Evidence of an antiinflammatory action. J. Clin. Endocrinol. Metab. 88, 4496–4501. 10.1210/jc.2002-021836 12970329

[B37] Danta C. C. (2021). SARS-CoV-2, hypoxia, and calcium signaling: The consequences and therapeutic options. ACS Pharmacol. Transl. Sci. 4, 400–402. 10.1021/acsptsci.0c00219 33615190 PMC7805596

[B38] Das A. Patra M. Dhangadamajhi G. (2021). Association of rs11549465 (C1772T) variant of hypoxia-inducible factor-1α with Covid-19 susceptibility. A population-based epidemiological study. Hum. Cell. 34, 1937–1940. 10.1007/s13577-021-00601-4 34426956 PMC8382105

[B39] Deshane J. Chen S. Caballero S. Grochot-Przeczek A. Was H. Li Calzi S. (2007). Stromal cell-derived factor 1 promotes angiogenesis via a heme oxygenase 1-dependent mechanism. J. Exp. Med. 204, 605–618. 10.1084/jem.20061609 17339405 PMC1855437

[B40] Deshmukh P. Unni S. Krishnappa G. Padmanabhan B. (2017). The Keap1- Nrf2 pathway: Promising therapeutic target to counteract ROS-mediated damage in cancers and neurodegenerative diseases. Biophys. Rev. 9, 41–56. 10.1007/s12551-016-0244-4 28510041 PMC5425799

[B41] Devaux C. A. Rolain J. M. Raoult D. (2020). ACE2 receptor polymorphism: Susceptibility to SARS-CoV-2, hypertension, multi-organ failure, and COVID-19 disease outcome. J. Microbiol. Immunol. Inf. 53, 425–435. 10.1016/j.jmii.2020.04.015 PMC7201239 32414646

[B42] Drozdovska S. B. Dosenko V. E. IIyin V. N. Filippov M. M. Kuzmina L. M. (2009). Allelic polymorphism of endothelial No-synthase (еNOS) Association with exercise-induced hypoxia adaptation. Balt. J. Health Phys. Act. 1, 13–19. 10.2478/v10131-009-0001-1

[B43] Duprez D. A. (2006). Role of the renin-angiotensin-aldosterone system in vascular remodeling and inflammation: A clinical review. J. Hypertens. 24, 983–991. 10.1097/01.hjh.0000226182.60321.69 16685192

[B44] Eigner D. Schilz D. (1999). *Ferula asa-foetida* and *Curcuma longa* in traditional medical treatment and diet in Nepal. J. Ethnopharmacol. 67, 1–6. 10.1016/s0378-8741(98)00234-7 10616954

[B45] Fan X. Ma L. Zhang Z. Li Y. Hao M. Zhao Z. (2018). Associations of high-altitude polycythemia with polymorphisms in PIK3CD and COL4A3 in Tibetan populations. Hum. Genomics 12 (1), 37. 10.1186/s40246-018-0169-z 30053909 PMC6062892

[B46] Fong G. H. Takeda K. (2008). Role and regulation of prolyl hydroxylase domain proteins. Cell. Death Differ. 15, 635–641. 10.1038/cdd.2008.10 18259202

[B47] Forth R. Montgomery H. (2003). ACE in COPD: A therapeutic target? Thorax 58, 556–558. 10.1136/thorax.58.7.556 12832663 PMC1746750

[B48] Foster G. E. Hanly P. J. Ahmed S. B. Beaudin A. E. Pialoux V. Poulin M. J. (2010). Intermittent hypoxia increases arterial blood pressure in humans through a renin-angiotensin system–dependent mechanism. Hypertension 56, 369–377. 10.1161/HYPERTENSIONAHA.110.152108 20625082

[B49] Freedman S. J. Sun Z. Y. J. Poy F. Kung A. L. Livingston D. M. Wagner G. (2002). Structural basis for recruitment of CBP/p300 by hypoxia-inducible factor-1 alpha. Proc. Natl. Acad. Sci. U. S. A. 99 (8), 5367–5372. 10.1073/pnas.082117899 11959990 PMC122775

[B50] Fricke-Galindo I. Falfan-Valencia R. (2021). Genetics insight for COVID-19 susceptibility and severity: A review. Front. Immunol. 12, 622176. 10.3389/fimmu.2021.622176 33868239 PMC8047200

[B51] Frutos R. Lopez Roig M. Serra-Cobo J. Devaux C. A. (2020). COVID-19: The conjunction of events leading to the Coronavirus pandemic and lessons to learn for future threats. Front. Med. 7, 223. 10.3389/fmed.2020.00223 PMC7235412 32574324

[B52] Fuglebjerg N. Jensen T. Hoyer N. Ryrsø C. Madsen B. Harboe Z. (2020). Silent hypoxia in patients with SARS CoV-2 infection before hospital discharge. Int. J. Infect. Dis. 99, 100–101. 10.1016/j.ijid.2020.07.014 32663601 PMC7836996

[B53] Fung M. L. Lam S. Y. Dong X. Chen Y. Leung P. S. (2002). Postnatal hypoxemia increases angiotensin II sensitivity and up-regulates AT1a angiotensin receptors in rat carotid body chemoreceptors. J. Endocrinol. 173, 305–313. 10.1677/joe.0.1730305 12010638

[B54] Fung M. L. (2014). The role of local renin-angiotensin system in arterial chemoreceptors in sleep-breathing disorders. Front. Physiol. 5, 336. 10.3389/fphys.2014.00336 25249981 PMC4155775

[B55] Gaber T. Dziurla R. Tripmacher R. Burmester G. R. Buttgereit F. (2005). Hypoxia inducible factor (HIF) in rheumatology: Low O2! See what HIF can do. Ann. Rheum. Dis. 64, 971–980. 10.1136/ard.2004.031641 15800008 PMC1755583

[B56] Gallo G. Calvez V. Savoia C. (2022a). Hypertension and COVID-19: Current evidence and perspectives. High. Blood Press. Cardiovasc. Prev. 29, 115–123. 10.1007/s40292-022-00506-9 35184271 PMC8858218

[B57] Gallo G. Volpe M. Savoia C. (2022b). Endothelial dysfunction in hypertension: Current concepts and clinical implications. Front. Med. 8, 798958. 10.3389/fmed.2021.798958 PMC8811286 35127755

[B58] Gautret P. Million M. Jarrot P. A. Camoin-Jau L. Colson P. Fenollar F. (2020). Natural history of COVID-19 and therapeutic options. Expert Rev. Clin. Immunol. 16 (12), 1159–1184. 10.1080/1744666X.2021.1847640 33356661

[B59] Gesang L. Liu G. Cen W. Qiu C. Zhuoma C. Zhuang L. (2002). Angiotensinconverting enzyme gene polymorphism and its association with essential hypertension in a Tibetan population. Hypertens. Res. 25, 481–485. 10.1291/hypres.25.481 12135330

[B60] Gladek I. Ferdin J. Horvat S. Calin G. A. Kunej T. (2017). HIF1A gene polymorphisms and human diseases: Graphical review of 97 association studies. Genes. Chromosom. Cancer 56 (6), 439–452. 10.1002/gcc.22449 28165644 PMC5395341

[B61] Griendling K. K. Minieri C. A. Ollerenshaw J. D. Alexander R. W. (1994). Angiotensin II stimulates NADH and NADPH oxidase activity in cultured vascular smooth muscle cells. Circ. Res. 74, 1141–1148. 10.1161/01.RES.74.6.1141 8187280

[B62] Guo X. Zhu Z. Zhang W. Meng X. Zhu Y. Han P. (2017). Nuclear translocation of HIF-1α induced by influenza A (H1N1) infection is critical to the production of proinflammatory cytokines: HIF-1α nuclear translocation induced by H1N1. Emerg. Microbes Infect. 6 (5), e39. 10.1038/emi.2017.21 28536432 PMC5520484

[B63] Gupta S. Aqrawal B. K. Goel R. K. Sehajpal P. K. (2009). Angiotensin-converting enzyme gene polymorphism in hypertensive rural population of Haryana. India Emerg. Trauma Shock 2 (3), 150–154. 10.4103/0974-2700.55323 PMC2776360 20009302

[B64] Gustafsson L. E. Leone A. M. Persson M. G. Wiklund N. P. Moncada S. (1991). Endogenous nitric oxide is present in the exhaled air of rabbits, Guinea pigs and humans. Biochem. Biophys. Res. Commun. 181, 852–857. 10.1016/0006-291x(91)91268-h 1721811

[B65] Hampl V. Herget J. Bıbova J. Banasova´ A. Huskova Z. Vanourkova´ Z. (2015). Intrapulmonary activation of the angiotensin-converting enzyme type 2/angiotensin 1–7/G-protein-coupled Mas receptor axis attenuates pulmonary hypertension in Ren-2 transgenic rats exposed to chronic hypoxia. Physiol. Res. 64, 25–38. 10.33549/physiolres.932861 25194138

[B66] Harrap S. B. Tzourio C. Cambien F. Poirier O. Raoux S. Chalmers J. (2003). The ACE gene I/D polymorphism is not associated with the blood pressure and cardiovascular benefits of ACE inhibition. Hypertension 42 (3), 297–303. 10.1161/01.HYP.0000088322.85804.96 12925557

[B67] Hassaniazad M. Inchehsablagh B. R. Kamali H. Tousi A. Eftekhar E. Jaafari M. R. (2020). The clinical effect of nano micelles containing curcumin as a therapeutic supplement in patients with COVID-19 and the immune responses balance changes following treatment: A structured summary of a study protocol for a randomised controlled trial. Trials 21, 876–922. 10.1186/s13063-020-04824-y 33092653 PMC7578586

[B68] Hatano N. Itoh Y. Suzuki H. Muraki Y. Hayashi H. Onozaki K. (2012). Hypoxia-inducible factor-1α (HIF1α) switches on transient receptor potential ankyrin repeat 1 (TRPA1) gene expression via a hypoxia response element-like motif to modulate cytokine release. J. Biol. Chem. 287 (38), 31962–31972. 10.1074/jbc.M112.361139 22843691 PMC3442528

[B69] Haug N. Geyrhofer L. Londei A. Dervic E. Desvars-Larrive A. Loreto V. (2020). Ranking the effectiveness of worldwide COVID-19 government interventions. Nat. Hum. Behav. 4, 1303–1312. 10.1038/s41562-020-01009-0 33199859

[B70] Hawkins K. E. Joy S. Delhove J. M. K. M. Kotiadis V. N. Fernandez E. Fitzpatrick L. M. (2016). NRF2 orchestrates the metabolic shift during induced pluripotent stem cell reprogramming. Cell. Rep. 14, 1883–1891. 10.1016/j.celrep.2016.02.003 26904936 PMC4785773

[B71] Herman J. Mori V. Bates J. H. T. Suki B. (2020). Modeling lung perfusion abnormalities to explain early COVID-19 hypoxemia. Nat. Commun. 11, 4883. 10.1038/s41467-020-18672-6 32985528 PMC7522238

[B72] Huamanı´ C. Velasquez L. Montes S. Miranda-Solis F. (2020). Propagation by COVID-19 at high altitude: Cusco case. Respir. Physiol. Neurobiol. 279, 103448. 10.1016/j.resp.2020.103448 32437878 PMC7207123

[B73] Huang C. Wang Y. Li X. Ren L. Zhao J. Hu Y. (2020). Clinical features of patients infected with 2019 novel coronavirus in Wuhan, China. Lancet 395 (10223), 497–506. 10.1016/S0140-6736(20)30183-5 31986264 PMC7159299

[B74] Imperio G. E. Lye P. Mughis H. Hamada H. Bloise E. Lye S. (2021). Hypoxia alters the expression of ACE2 and TMPRSS2 SARS-CoV-2 cell entry mediators in hCMEC/D3 brain endothelial cells. Microvasc. Res. 138, 104232. 10.1016/j.mvr.2021.104232 34416267 PMC8372440

[B75] Ioannidis J. P. A. (2020). Global perspective of COVID‐19 epidemiology for a full‐cycle pandemic. Eur. J. Clin. Invest. 50 (12), e13423. 10.1111/eci.13423 33026101 PMC7646031

[B76] Jahani M. Dokaneheifard S. Mansouri K. (2020). Hypoxia: A key feature of COVID-19 launching activation of HIF-1 and cytokine storm. J. Inflamm. 17, 33. 10.1186/s12950-020-00263-3 PMC7594974 33139969

[B77] Jayatunga M. K. P. Thompson S. McKee T. C. Chan M. C. Reece K. M. Hardy A. P. (2015). Inhibition of the HIF1α-p300 interaction by quinone- and indandione-mediated ejection of structural Zn(II). Eur. J. Med. Chem. 94, 509–516. 10.1016/j.ejmech.2014.06.006 25023609 PMC4277744

[B78] Jeddi F. Soozangar N. Sadeghi M. R. Somi M. H. Samadi N. (2017). Contradictory roles of Nrf2/Keap1 signaling pathway in cancer prevention/promotion and chemoresistance. DNA Repair (Amst.) 54, 13–21. 10.1016/j.dnarep.2017.03.008 28415030

[B79] Joshi S. Wollenzien H. Leclerc E. Jarajapu Y. P. (2019). Hypoxic regulation of angiotensin-converting enzyme 2 and Mas receptor in human CD34(+) cells. J. Cell. Physiol. 234, 20420–20431. 10.1002/jcp.28643 30989646 PMC6660366

[B80] Julian C. G. (2017). Epigenomics and human adaptation to high altitude. J. Appl. Physiol. 123, 1362–1370. 10.1152/japplphysiol.00351.2017 28819001 PMC6157641

[B81] Julian C. G. Moore L. G. (2019). Human genetic adaptation to high altitude: Evidence from the andes. Genes. 10, 150. 10.3390/genes10020150 PMC6410003 30781443

[B82] Kallio P. J. Okamoto K. O'Brien S. Carrero P. Makino Y. Tanaka H. (1998). Signal transduction in hypoxic cells: Inducible nuclear translocation and recruitment of the CBP/p300 coactivator by the hypoxia-inducible factor-1alpha. EMBO J. 17 (22), 6573–6586. 10.1093/emboj/17.22.6573 9822602 PMC1171004

[B83] Kim H. O. Jo Y. H. Lee J. Lee S. S. Yoon K. S. (2008). The C1772T genetic polymorphism in human HIF-1alpha gene associates with expression of HIF-1alpha protein in breast cancer. Oncol. Rep. 20, 1181–1187. 10.3892/or_00000127 18949419

[B84] Kim S. H. Jeong J. W. Park J. A. Lee J. W. Seo J. H. Jung B. K. (2007). Regulation of the HIF-1α stability by histone deacetylases. Oncol. Rep. 17 (3), 647–651. 10.3892/or.17.3.647 17273746

[B85] Kobayashi E. H. Suzuki T. Funayama R. Nagashima T. Hayashi M. Sekine H. (2016). Nrf2 suppresses macrophage inflammatory response by blocking proinflammatory cytokine transcription. Nat. Commun. 7, 11624. 10.1038/ncomms11624 27211851 PMC4879264

[B86] Kung A. L. Wang S. Klco J. M. Kaelin W. G. Livingston D. M. (2001). Suppression of tumor growth through disruption of hypoxia-inducible transcription. Nat. Med. 6 (12), 1335–1340. 10.1038/82146 11100117

[B87] Lachance G. Uniacke J. Audas T. E. Holterman C. E. Franovic A. Payette J. (2014). DNMT3a epigenetic program regulates the HIF-2α oxygen-sensing pathway and the cellular response to hypoxia. Proc. Natl. Acad. Sci. U. S. A. 111, 7783–7788. 10.1073/pnas.1322909111 24817692 PMC4040594

[B88] Lacher S. E. Levings D. C. Freeman S. Slattery M. (2018). Identification of a functional antioxidant response element at the HIF1A locus? Redox Biol. 19, 401–411. 10.1016/j.redox.2018.08.014 30241031 PMC6146589

[B89] Lam S. Y. Fung M. L. Leung P. S. (2004). Regulation of the angiotensinconverting enzyme activity by a time-course hypoxia in the carotid body. J. Appl. Physiol. 96, 809–813. 10.1152/japplphysiol.00684.2003 14527966

[B90] Lee J. W. Bae S. H. Jeong J. W. Kim S. H. Kim K. W. (2004). Hypoxia-inducible factor (HIF-1)alpha: Its protein stability and biological functions. Exp. Mol. Med. 36 (1), 1–12. 10.1038/emm.2004.1 15031665

[B91] Lee J. W. Ko J. Eltzchig H. K. Eltzschig H. K. (2019). Hypoxia signaling in human diseases and therapeutic targets. Exp. Mol. Med. 51, 1–13. 10.1038/s12276-019-0235-1 PMC6586801 31221962

[B92] Lester S. N. Li K. (2014). Toll-like receptors in antiviral innate immunity. J. Mol. Biol. 426, 1246–1264. 10.1016/j.jmb.2013.11.024 24316048 PMC3943763

[B93] Leung P. S. Lam S. Y. Fung M. L. (2000). Chronic hypoxia upregulates the expression and function of AT(1) receptor in rat carotid body. J. Endocrinol. 167, 517–524. 10.1677/joe.0.1670517 11115779

[B94] Liang D. Kong X. Sang N. (2006). Effects of histone deacetylase inhibitors on HIF-1. Cell. Cycle 5 (21), 2430–2435. 10.4161/cc.5.21.3409 17102633 PMC4505804

[B95] Lin J. K. (2007). Molecular targets of curcumin. Adv. Exp. Med. Biol. 595, 227–243. 10.1007/978-0-387-46401-5_10 17569214

[B96] Liu S. S. Wang H. Y. Tang J. M. Zhou X. M. (2013). Hypoxia-induced collagen synthesis of human lung fibroblasts by activating the angiotensin system. Int. J. Mol. Sci. 14, 24029–24045. 10.3390/ijms141224029 24336063 PMC3876092

[B97] Lyer N. V. Leung S. W. Semenza G. L. (1998). The human hypoxia-inducible factor 1alpha gene: HIF1A structure and evolutionary conservation. Genomics 52, 159–165. 10.1006/geno.1998.5416 9782081

[B98] Mahabeleshwar G. H. Qureshi M. A. Takami Y. Sharma N. Lingrel J. B. Jain M. K. (2012). A myeloid hypoxia-inducible factor 1-Krüppel-like factor 2 pathway regulates gram-positive endotoxin- mediated sepsis. J. Biol. Chem. 287, 1448–1457. 10.1074/jbc.M111.312702 22110137 PMC3256857

[B99] Marshall R. P. Webb S. Bellingan G. J. Montgomery H. E. Chaudhari B. Mc Arthur R. J. (2002). Angiotensin converting enzyme insertion/deletion polymorphism is associated with susceptibility and outcome in acute respiratory distress syndrome. Am. J. Respir. Crit. Care Med. 166, 646–650. 10.1164/rccm.2108086 12204859

[B100] Matsuura H. Ichiki T. Ikeda J. Takeda K. Miyazaki R. Hashimoto T. (2011). Inhibition of prolyl hydroxylase domain-containing protein downregulates vascular angiotensin II type 1 receptor. Hypertension 58, 386–393. 10.1161/HYPERTENSIONAHA.110.167106 21825224

[B101] Maurya V. K. Kumar S. Prasad A. K. Bhatt M. L. B. Saxena S. K. (2020). Structure-based drug designing for potential antiviral activity of selected natural products from ayurveda against SARS-CoV-2 spike glycoprotein and its cellular receptor. Virusdisease 31, 179–193. 10.1007/s13337-020-00598-8 32656311 PMC7245990

[B102] Maxwell P. H. Pugh C. W. Maher E. R. Ratcliffe P. J. (1993). Inducible operation of the erythropoietin 3' enhancer in multiple cell ^l^ines: Evidence for a widespread oxygen-sensing mechanism. Proc. Natl. Acad. Sci. U. S. A. 90 (6), 2423–2427. 10.1073/pnas.90.6.2423 8460154 PMC46099

[B103] Maxwell P. H. Wiesener M. S. Chang G. W. Clifford S. C. Vaux E. C. Cockman M. E. (1999). The tumour suppressor protein VHL target^s^ hypoxia-inducible factors for oxygen-dependent proteolysis. Nature 399 (6733), 271–275. 10.1038/20459 10353251

[B104] McClendon J. Jansing N. L. Redente E. F. Gandjeva A. Ito Y. Colgan S. P. (2017). Hypoxia-inducible factor 1α signaling promotes repair of the alveolar epithelium after acute lung injury. Am. J. Pathol. 187, 1772–1786. 10.1016/j.ajpath.2017.04.012 28618253 PMC5530913

[B105] McFarlane S. Nicholl M. J. Sutherland J. S. Preston C. M. (2011). Interaction of the human cytomegalovirus particle with the host cell induces hypoxia-inducible factor 1 alpha. Virology 414, 83–90. 10.1016/j.virol.2011.03.005 21481907

[B106] Metzen E. Stiehl D. P. Doege K. Marxsen J. H. Hellwig-Bürgel T. Jelkmann W. (2005). Regulation of the prolyl hydroxylase domain protein 2 (phd2/egln-1) gene: Identification of a functional hypoxia-responsive element. Biochem. J. 387, 711–717. 10.1042/BJ20041736 15563275 PMC1135001

[B107] Michel G. Minet E. Ernest I. Durant F. Remacle J. Michiels C. (1999). Molecular modeling of the hypoxia-inducible factor-1 (HIF-1). Theor. Chem. Accounts Theory Comput. Model. 101, 51–56. 10.1007/s002140050405

[B108] Moore L. G. Shriver M. Bemis L. Hickler B. Wilson M. Brutsaert T. (2004). Maternal adaptation to high-altitude pregnancy: An experiment of nature - a review. Placenta 25, S60–S71. 10.1016/j.placenta.2004.01.008 15033310

[B109] Moore L. G. Zamudio S. Zhuang J. Droma T. Shohet R. V. (2002). Analysis of the myoglobin gene in Tibetans living at high altitude. High. Alt. Med. Biol. 3, 39–47. 10.1089/152702902753639531 12006163

[B110] Mori Y. Takahashi N. Kurokawa T. Kiyonaka S. (2017). TRP channels in oxygen physiology: Distinctive functional properties and roles of TRPA1 in O2 sensing. Proc. Jpn. Acad. Ser. B Phys. Biol. Sci. 93, 464–482. 10.2183/pjab.93.028 PMC5713176 28769017

[B111] Morrell N. W. Higham M. A. Phillips P. G. Shakur B. H. Robinson P. J. Beddoes R. J. (2005). Pilot study of losartan for pulmonary hypertension in chronic obstructive pulmonary disease. Respir. Res. 6, 88. 10.1186/1465-9921-6-88 16060962 PMC1198258

[B112] Morrell N. W. Morris K. G. Stenmark K. R. (1995). Role of angiotensin converting enzyme and angiotensin II in development of hypoxic pulmonary hypertension. Am. J. Physiol. 269, H1186–H1194. 10.1152/ajpheart.1995.269.4.H1186 7485548

[B113] Motohashi N. Vanam A. Gollapudi R. (2020). *In silico* study of Curcumin and Folic Acid as potent inhibitors of human Transmembrane protease serine 2 in the treatment of COVID-19. INNOSC Theranostics Pharmacol. Sci. 3, 3–9. 10.36922/itps.v3i2.935

[B114] Moulin S. Arnaud C. Bouyon S. Pépin J. L. Godin-Ribuot D. Elise Belaidi E. (2020). Curcumin prevents chronic intermittent hypoxia-induced myocardial injury. Ther. Adv. Chronic Dis. 11, 2040622320922104–2040622320922113. 10.1177/2040622320922104 32637058 PMC7315663

[B115] Munzenmaier D. H. Greene A. S. (1996). Opposing actions of angiotensin II on microvascular growth and arterial blood pressure. Hypertension 27, 760–765. 10.1161/01.hyp.27.3.760 8613237

[B116] Nagarajan Y. Rychkov G. Y. Peet D. J. (2017). Modulation of TRP channel activity by hydroxylation and its therapeutic potential. Pharmaceuticals 10 (2), 35. 10.3390/ph10020035 PMC5490392 28346371

[B117] Nangaku M. Fujita T. (2008). Activation of the renin-angiotensin system and chronic hypoxia of the kidney. Hypertens. Res. 31 (2), 175–184. 10.1291/hypres.31.175 18360035

[B118] Nitsure M. Sarangi B. Shankar G. H. Reddy V. S. Walimbe A. Sharma V. (2020). Mechanisms of hypoxia in COVID-19 patients: A pathophysiologic reflection. Indian J. Crit. Care Med. 24 (10), 967–970. 10.5005/jp-journals-10071-23547 33281323 PMC7689135

[B119] Nizet V. Johnson R. S. (2009). Interdependence of hypoxic and innate immune responses. Nat. Rev. Immunol. 9 (9), 609–617. 10.1038/nri2607 19704417 PMC4343208

[B120] Oarhe C. I. Dang V. Dang M. Nguyen H. Gopallawa I. Gewolb I. H. (2015). Hyperoxia downregulates angiotensin-converting enzyme-2 in human fetal lung fibroblasts. Pediatr. Res. 77, 656–662. 10.1038/pr.2015.27 25665060 PMC5119454

[B121] Ollerenshaw M. Page T. Hammonds J. Demaine A. (2004). Polymorphisms in the hypoxia inducible factor-1alpha gene (HIF1A) are associated with the renal cell carcinoma phenotype. Cancer Genet. cytogenet. 153 (2), 122–126. 10.1016/j.cancergencyto.2004.01.014 15350301

[B122] Osman I. O. Melenotte C. Brouqui P. Million M. Lagier J-C. Parola P. (2021). Expression of ACE2, soluble ACE2, angiotensin I, angiotensin II and angiotensin-(1-7) is modulated in COVID-19 patients. Front. Immunol. 12, 625732. 10.3389/fimmu.2021.625732 34194422 PMC8236950

[B123] Othman H. Bouslama Z. Brandenburg J. T. da Rocha J. Hamdi Y. Ghedira K. (2020). Interaction of the spike protein RBD from SARS-CoV-2 with ACE2: Similarity with SARS-CoV, hot-spot analysis and effect of the receptor polymorphism. Biochem. Biophys. Res. Commun. 527, 702–708. 10.1016/j.bbrc.2020.05.028 32410735 PMC7221370

[B124] Ouyang S. Yao Y. H. Zhang Z. M. Liu J. S. Xiang H. (2019). Curcumin inhibits hypoxia inducible factor-1α-induced inflammation and apoptosis in macrophages through an ERK dependent pathway. Eur. Rev. Med. Pharmacol. Sci. 23, 1816–1825. 10.26355/eurrev_201902_17145 30840308

[B125] Paciello F. Fetoni A. R. Mezzogori D. Rolesi R. Di Pino A. Paludetti G. (2020). The dual role of curcumin and ferulic acid in counteracting chemoresistance and cisplatin-induced ototoxicity. Sci. Rep. 10, 1063. 10.1038/s41598-020-57965-0 31974389 PMC6978317

[B126] Pawar K. S. Mastud R. N. Pawar S. K. Pawar S. S. Bhoite R. R. Bhoite R. R. (2021). Oral curcumin with piperine as adjuvant therapy for the treatment of COVID‐19: A randomized clinical trial. Front. Pharmacol. 12, 669362. 10.3389/fphar.2021.669362 34122090 PMC8193734

[B127] Peng T. Du S. Y. Son M. Diamond B. (2021). HIF-1α is a negative regulator of interferon regulatory factors: Implications for interferon production by hypoxic monocytes. Proc. Natl. Acad. Sci. U. S. A. 118 (26), e2106017118. 10.1073/pnas.2106017118 34108245 PMC8256008

[B128] Pore N. Jiang Z. Gupta A. Cerniglia G. Kao G. D. Maity A. (2006). EGFR tyrosine kinase inhibitors decrease VEGF expression by both Hypoxia-Inducible Factor (HIF)-1–Independent and HIF-1–dependent mechanisms. Cancer Res. 66 3197–3204. 10.1158/0008-5472.CAN-05-3090 16540671

[B129] Potteti H. R. Noone P. M. Tamatam C. R. Ankireddy A. Noel S. Rabb H. (2021). Nrf2 mediates hypoxia-inducible HIF1α activation in kidney tubular epithelial cells. Am. J. Physiol. Ren. Physiol. 320, F464–F474. 10.1152/ajprenal.00501.2020 PMC7988808 33491566

[B130] Prabhakar N. R. Peng Y. J. (2004). Peripheral chemoreceptors in health and disease. J. Appl. Physiol. 96, 359–366. 10.1152/japplphysiol.00809.2003 14660497

[B131] Prieto-Fernandez E. Egia-Mendikute L. Vila-Vecilla L. Lee S. Y. Bosch A. Barreira-Manrique A. (2021). Hypoxia reduces cell attachment of SARS-CoV-2 spike protein by modulating the expression of ACE2 and heparan sulfate. bioRxiv. preprint. 10.1101/2021.01.09.426021 PMC8183554 34013835

[B132] Pun M. Turner R. Strapazzon G. Brugger H. Swenson E. R. (2020). Lower incidence of COVID-19 at high altitude: Facts and confounders. High. Alt. Med. Biol. 21 (3), 217–222. 10.1089/ham.2020.0114 32716669

[B133] Qin X. Chen H. Tu L. Ma Y. Liu N. Zhang H. (2021). Potent inhibition of HIF1α and p300 interaction by a constrained peptide derived from CITED2. J. Med. Chem. 64 (18), 13693–13703. 10.1021/acs.jmedchem.1c01043 34472840

[B134] Qiu Y. Zhao Y. B. Wang Q. Li J. Y. Zhou Z. J. Liao C. H. (2020). Predicting the angiotensin converting enzyme 2 (ACE2) utilizing capability as the receptor of SARS-CoV-2. Microbes Infect. 22, 221–225. 10.1016/j.micinf.2020.03.003 32199943 PMC7156207

[B135] Raf H. (1991). “The renin-angiotensin-aldosterone system during hypoxia,” in Response and adaptation to hypoxia. Editor Lahiri S. (Springer, New York: American Physiology Society), 211–212. Chapter 20. 10.1007/978-1-4614-7574-3_20

[B136] Rahman A. Tabassum T. Araf Y. Al Nahid A. Ullah M. A. Jakir Hosen M. (2021). Silent hypoxia in COVID-19: Pathomechanism and possible management strategy. Mol. Biol. Rep. 48 (4), 3863–3869. 10.1007/s11033-021-06358-1 33891272 PMC8062941

[B137] Rajagopalan S. Kurz S. Munzel T. Tarpey M. Freeman B. A. Griendling K. K. (1996). Angiotensin II-mediated hypertension in the rat increases vascular superoxide production via membrane NADH/NADPH oxidase activation. Contribution to alterations of vasomotor tone. J. Clin. Invest. 97, 1916–1923. 10.1172/JCI118623 8621776 PMC507261

[B138] Ratcliffe P. J. (2013). Oxygen sensing and hypoxia signalling pathways in animals: The implications of physiology for cancer. J. Physiol. 591 (8), 2027–2042. 10.1113/jphysiol.2013.251470 23401619 PMC3634517

[B139] Rattis B. A. C. Ramos S. G. Celes M. R. N. (2021). Curcumin as a potential treatment for COVID-19. Front. Pharmacol. 12, 675287. 10.3389/fphar.2021.675287 34025433 PMC8138567

[B140] Ripoli M. D'Aprile A. Quarato G. Sarasin-Filipowicz M. Gouttenoire J. Scrima R. (2010). Hepatitis C virus-linked mitochondrial dysfunction promotes hypoxia-inducible factor 1α-mediated glycolytic adaptation. J. Virol. 84 (1), 647–660. 10.1128/JVI.00769-09 19846525 PMC2798449

[B141] Rysz S. Al-Saadi J. Sjöström A. Farm M. Campoccia Jalde F. Plattén M. (2021). COVID-19 pathophysiology may be driven by an imbalance in the renin-angiotensin-aldosterone system. Nat. Commun. 12, 2417. 10.1038/s41467-021-22713-z 33893295 PMC8065208

[B142] Saber‐Moghaddam N. Salari S. Hejazi S. Amini M. Taherzadeh Z. Eslami S. (2021). Oral nano‐curcumin formulation efficacy in management of mild to moderate hospitalized coronavirus disease‐19 patients: An open label nonrandomized clinical trial. Phytotherapy Res. 34 (12), 2616–2623. 10.1002/ptr.7004 33389761

[B143] Santos S. A. D. Andrade D. R. D. Júnior (2017). HIF-1alpha and infectious diseases: A new frontier for the development of new therapies. Rev. Inst. Med. Trop. Sao Paulo 59, e92–10. 10.1590/S1678-9946201759092 29267600 PMC5738998

[B144] Sarighieh M. A. Montazeri V. Shadboorestan A. Ghahremani M. H. Ostad S. N. (2020). The inhibitory effect of curcumin on hypoxia inducer factors (hifs) as a regulatory factor in the growth of tumor cells in breast cancer stem-like cells. Drug Res. 70, 512–518. 10.1055/a-1201-2602 32961574

[B145] Scherrer U. Vollenweider L. Delabays A. Savcic M. Eichenberger U. Kleger G. R. (1996). Inhaled nitric oxide for high-altitude pulmonary edema. N. Engl. J. Med. 334, 624–629. 10.1056/NEJM199603073341003 8592525

[B146] Semenza G. L. (2000a). HIF-1: Mediator of physiological and pathophysiological responses to hypoxia. J. Appl. Physiol. 88, 1474–1480. 10.1152/jappl.2000.88.4.1474 10749844

[B147] Semenza G. L. (2000b). Oxygen-regulated transcription factors and their role in pulmonary disease. Respir. Res. 1 (3), 159–162. 10.1186/rr27 11667980 PMC59554

[B148] Serebrovska Z. O. Chong E. Y. Serebrovska T. V. Tumanovska L. V. Xi L. (2020). Hypoxia, HIF-1α, and COVID-19: From pathogenic factors to potential therapeutic targets. Acta Pharmacol. Sin. 41, 1539–1546. 10.1038/s41401-020-00554-8 33110240 PMC7588589

[B149] Seta K. A. Yuan Y. Spicer Z. Lu G. Bedard J. Ferguson T. K. (2004). The role of calcium in hypoxia-induced signal transduction and gene expression. Cell. Calcium 36 (3-4), 331–340. 10.1016/j.ceca.2004.02.006 15261489

[B150] Shanmugarajan D. Prabitha P. Prashantha Kumar B. R. Suresh B. (2020). Curcumin to inhibit binding of spike glycoprotein to ACE2 receptors: Computational modelling, simulations, and ADMET studies to explore curcuminoids against novel SARS-CoV-2 targets. RSC Adv. 10, 31385–31399. 10.1039/d0ra03167d 35520671 PMC9056388

[B151] Shaw P. Chattopadhyay A. (2019). Nrf2–ARE signaling in cellular protection: Mechanism of action and the regulatory mechanisms. J. Cell. Physiol. 235, 3119–3130. 10.1002/jcp.29219 31549397 PMC13482834

[B152] Shimizu K. Sunagawa Y. Funamoto M. Wakabayashi H. Genpei M. Miyazaki Y. (2020). The synthetic curcumin analogue GO-Y030 effectively suppresses the development of pressure overload-induced heart failure in mice. Sci. Rep. 10, 7172. 10.1038/s41598-020-64207-w 32346115 PMC7188884

[B153] Simonson T. S. Yang Y. Huff C. D. Yun H. Qin G. Witherspoon D. J. (2010). Genetic evidence for high-altitude adaptation in Tibet. Science 329, 72–75. 10.1126/science.1189406 20466884

[B154] Stobdan T. Ali Z. Pervez Khan A. Nejatizadeh A. Ram R. Thinlas T. (2011). Polymorphisms of renin-angiotensin system genes as a risk factor for high-altitude pulmonary oedema. J. Renin. Angiotensin. Aldosterone. Syst. 12 (2), 93–101. 10.1177/1470320310387177 21393362

[B155] Sunagawa Y. Morimoto T. Wada H. Takaya T. Katanasaka Y. Kawamura T. (2011). A natural p300-specific Histone Acetyltransferase inhibitor, curcumin, in addition to Angiotensin-Converting Enzyme inhibitor, exerts beneficial effects on left ventricular systolic function after myocardial infarction in rats. Circ. J. 75 (9), 2151–2159. 10.1253/circj.CJ-10-1072 21737953

[B156] Suryamohan K. Diwanji D. Stawiski E. W. Gupta R. Miersch S. Liu J. (2021). Human ACE2 receptor polymorphisms and altered susceptibility to SARS-CoV-2. Commun. Biol. 4, 475. 10.1038/s42003-021-02030-3 33846513 PMC8041869

[B157] Suzuki K. Kizaki T. Hitomi Y. Nukita M. Kimoto K. Miyazawa N. (2003). Genetic variation in hypoxia-inducible factor 1alpha and its possible association with high altitude adaptation in Sherpas. Med. Hypotheses 61, 385–389. 10.1016/s0306-9877(03)00178-6 12944107

[B158] Tahmasebi S. El-Esawi M. A. Mahmoud Z. H. Timoshin A. Valizadeh H. Roshangar L. (2020). Immunomodulatory Effects of Nanocurcumin on Th17 cell responses in mild and severe COVID-19 patients. J. Cell. Physiol. 236, 5325–5338. 10.1002/jcp.30233 33372280

[B159] Takahashi S. Nakamura Y. Nishijima T. Sakurai S. Inoue H. (2005). Essential roles of angiotensin II in vascular endothelial growth factor expression in sleep apnea syndrome. Respir. Med. 99, 1125–1131. 10.1016/j.rmed.2005.02.027 16085213

[B160] Tian M. Liu W. Li X. Zhao P. Shereen M. A. Zhu C. (2021). HIF-1α promotes SARS-CoV-2 infection and aggravates inflammatory responses to COVID-19. Signal Transduct. Target. Ther. 6, 308. 10.1038/s41392-021-00726-w 34408131 PMC8371950

[B161] Tobin M. Laghi F. Jubran A. (2020). Why COVID-19 silent hypoxemia Is baffling to physicians. Am. J. Respir. Crit. Care Med. 202 (3), 356–360. 10.1164/rccm.202006-2157CP 32539537 PMC7397783

[B162] Toh R. K. Warfel N. A. (2017). Strange bedfellows: Nuclear factor, erythroid 2-like 2 (Nrf2) and hypoxia-inducible factor 1 (HIF-1) in tumor hypoxia. Antioxidants (Basel) 6 (2), 27. 10.3390/antiox6020027 PMC5488007 28383481

[B163] Trigo‐Gutierrez J. K. Vega‐Chacon Y. Soares A. B. Mima E. G. O. (2021). Antimicrobial activity of curcumin in nanoformulations: A comprehensive review. Int. J. Mol. Sci. 22 (13), 7130. 10.3390/ijms22137130 34281181 PMC8267827

[B164] Trisciuoglio D. Gabellini C. Desideri M. Ragazzoni Y. De Luca T. Ziparo E. (2011). Involvement of BH4 domain of bcl-2 in the regulation of HIF-1-mediated VEGF expression in hypoxic tumor cells. Cell. Death Differ. 18, 1024–1035. 10.1038/cdd.2010.175 21233846 PMC3131942

[B165] Valizadeh H. Abdolmohammadi-vahid S. Danshina S. Ziya Gencer M. Ammari A. Sadeghi A. (2020). Nano-curcumin therapy, a promising method in modulating inflammatory cytokines in COVID-19 patients. Int. Immunopharmacol. 89, 107088. 10.1016/j.intimp.2020.107088 33129099 PMC7574843

[B166] Valverde G. Zhou H. Lippold S. de Filippo C. Tang K. Lopez H. (2015). A novel candidate region for genetic adaptation to high altitude in Andean populations. PLoS One 10 (5), e0125444. 10.1371/journal.pone.0125444 25961286 PMC4427407

[B167] Vishvakarma N. K. Kumar A. Singh S. M. (2011). Role of curcumindependent modulation of tumor microenvironment of a murine T cell lymphoma in altered regulation of tumor cell survival. Toxicol. Appl. Pharmacol. 252, 298–306. 10.1016/j.taap.2011.03.002 21397623

[B168] Vyas A. Dandawate P. Padhye S. Ahmad A. Sarkar F. (2013). Perspectives on new synthetic curcumin analogs and their potential anticancer properties. Curr. Pharm. Des. 19 (11), 2047–2069. 10.2174/1381612811319110007 23116312 PMC3788358

[B169] Wakahara S. Konoshita T. Mizuno S. Motomura M. Aoyama C. Makino Y. (2007). Synergistic expression of angiotensin-converting enzyme (ACE) and ACE2 in human renal tissue and confounding effects of hypertension on the ACE to ACE2 Ratio. Endocrinology 148 (5), 2453–2457. 10.1210/en.2006-1287 17303661

[B170] Wang F. Zhang R. Wu X. Hankinson O. (2010). Roles of coactivators in hypoxic induction of the erythropoietin gene. PLoS One 5 (4), e10002. 10.1371/journal.pone.0010002 20368990 PMC2848849

[B171] Wang G. L. Jiang B. H. Rue E. A. Semenza G. L. (1995). Hypoxiainducible factor 1 is a basic-helix-loop-helixPAS heterodimer regulated by cellular O_2_ tension. Proc. Natl. Acad. Sci. U. S. A. 92 (12), 5510–5514. 10.1073/pnas.92.12.5510 7539918 PMC41725

[B172] Wang L. Tang Y. Chen Y. (2018). HIF1AHIF1A gene rs10873142 polymorphism is associated with risk of chronic obstructive pulmonary disease in a Chinese han population: A case-control study. Biosci. Rep. 38 (2), BSR20171309. 10.1042/BSR20171309 29339421 PMC5843754

[B173] Wei C. Wang H. Liu G. Zhao F. Kijas J. W. Ma Y. (2016). Genome-wide analysis reveals adaptation to high altitudes in Tibetan sheep. Sci. Rep. 6, 26770. 10.1038/srep26770 27230812 PMC4882523

[B174] Wing P. A. C. Keeley T. P. Zhuang X. Lee J. Y. Prange-Barczynska M. Tsukuda S. (2021). Hypoxic and pharmacological activation of HIF inhibits SARS-CoV-2 infection of lung epithelial cells. Cell. Rep. 35, 109020. 10.1016/j.celrep.2021.109020 33852916 PMC8020087

[B175] Wolf G. Schroeder R. Stahl R. A. K. (2004). Angiotensin II induces hypoxia-inducible factor-1 alpha in PC 12 cells through a posttranscriptional mechanism: Role of AT2 receptors. Am. J. Nephrol. 24 (4), 415–421. 10.1159/000080086 15308873

[B176] Wu T. Wang X. Wei C. Cheng H. Wang X. Li Y. (2005). Hemoglobin levels in qinghai-tibet: Different effects of gender for Tibetans vs. Han. J. Appl. Physiol. 98, 598–604. 10.1152/japplphysiol.01034.2002 15258131

[B177] Xi A. Zhuo M. Dai J. Ding Y. Ma X. Ma X. (2020). Epidemiological and clinical characteristics of discharged patients infected with SARS-CoV-2 on the Qinghai plateau. J. Med. Virol. 92 (11), 2528–2535. 10.1002/jmv.26032 32437017 PMC7280617

[B178] Xue Q. Dasgupta C. Chen M. Zhang L. (2011). Foetal hypoxia increases cardiac AT2R expression and subsequent vulnerability to adult ischaemic injury. Cardiovasc. Res. 89, 300–308. 10.1093/cvr/cvq303 20870653 PMC3020132

[B179] Yamamoto N. Nishida N. Yamamoto R. Gojobori T. Shimotohno K. Mizokami M. (2021). Angiotensin–converting enzyme (ACE) 1 gene polymorphism and phenotypic expression of COVID-19 symptoms. Genes. 12, 1572. 10.3390/genes12101572 34680966 PMC8535484

[B180] Yan R. Zhang Y. Li Y. Xia L. Guo Y. Zhou Q. (2020). Structural basis for the recognition of SARS-CoV-2 by full-length human ACE2. Science 367 (6485), 1444–1448. 10.1126/science.abb2762 32132184 PMC7164635

[B181] Yi X. Liang Y. Huerta-Sanchez E. Jin X. Ping Guo Z. X. Pool J. E. (2010). Sequencing of 50 human exomes reveals adaptation to high altitude. Science 329, 75–78. 10.1126/science.1190371 20595611 PMC3711608

[B182] Yoon H. Lim J. H. Cho C. H. Huang L. E. Park J. W. (2011). CITED2 controls the hypoxic signaling by snatching p300 from the two distinct activation domains of HIF-1α. Biochim. Biophys. Acta 1813, 2008–2016. 10.1016/j.bbamcr.2011.08.018 21925214

[B183] Zakheim R. M. Molteni A. Mattioli L. Park M. (1976). Plasma angiotensin II levels in hypoxic and hypovolemic stress in unanesthetized rabbits. J. Appl. Physiol. 41, 462–465. 10.1152/jappl.1976.41.4.462 985386

[B184] Zeng J. Peng S. Lei Y. Huang J. Guo Y. Zhang X. (2020). Clinical and imaging features of COVID-19 patients: Analysis of data from high-altitude areas. J. Infect. 80, e34–e36. 10.1016/j.jinf.2020.03.026 32275925 PMC7141460

[B185] Zhang R. Wu Y. Zhao M. Liu C. Zhou L. Shen S. (2009). Role of HIF-1alpha in the regulation ACE and ACE2 expression in hypoxic human pulmonary artery smooth muscle cells. Am. J. Physiol. Lung Cell. Mol. Physiol. 297, L631–L640. 10.1152/ajplung.90415.2008 19592460

[B186] Zhou H. Beevers C. S. Huang S. (2011). The targets of curcumin. Curr. Drug Targets 12 (3), 332–347. 10.2174/138945011794815356 20955148 PMC3025067

[B187] Zhou P. Yang X. L. Wang X. G. Hu B. Zhang L. Zhang W. (2020). A pneumonia outbreak associated with a new coronavirus of probable bat origin. Nature 579, 270–273. 10.1038/s41586-020-2012-7 32015507 PMC7095418

[B188] Zhu N. Zhang D. Wang W. Li X. Yang B. Song J. (2020). A Novel Coronavirus from patients with pneumonia in China, 2019. N. Engl. J. Med. 382, 727–733. 10.1056/NEJMoa2001017 31978945 PMC7092803

[B189] Zhu Q. Wang Z. Xia M. Li P. L. Van Tassell B. W. Abbate A. (2011). Silencing of hypoxia-inducible factor-1α gene attenuated angiotensin II-induced renal injury in Sprague-Dawley rats. Hypertension 58, 657–664. 10.1161/hypertensionaha.111.177626 21896938 PMC3174356

[B190] Zimna A. Kurpisz M. (2015). Hypoxia-inducible factor-1 in physiological and pathophysiological angiogenesis: Applications and therapies. Biomed. Res. Int. 2015, 549412. 10.1155/2015/549412 26146622 PMC4471260

